# Overexpression of the Eggplant (*Solanum melongena*) NAC Family Transcription Factor S*mNAC* Suppresses Resistance to Bacterial Wilt

**DOI:** 10.1038/srep31568

**Published:** 2016-08-16

**Authors:** Chen Na, Wu Shuanghua, Fu Jinglong, Cao Bihao, Lei Jianjun, Chen Changming, Jiang Jin

**Affiliations:** 1 College of Horticulture, South Agricultural University, Guangzhou City, 510642, P.R. China

## Abstract

Bacterial wilt (BW) is a serious disease that affects eggplant (*Solanum melongena*) production. Although resistance to this disease has been reported, the underlying mechanism is unknown. In this study, we identified a NAC family transcription factor (*SmNAC*) from eggplant and characterized its expression, its localization at the tissue and subcellular levels, and its role in BW resistance. To this end, transgenic eggplant lines were generated in which the expression of *SmNAC* was constitutively up regulated or suppressed using RNAi. The results indicated that overexpression of *SmNAC* decreases resistance to BW. Moreover, *SmNAC* overexpression resulted in the reduced accumulation of the plant immune signaling molecule salicylic acid (SA) and reduced expression of *ICS1* (a gene that encode isochorismate synthase 1, which is involved in SA biosynthesis). We propose that reduced SA content results in increased bacterial wilt susceptibility in the transgenic lines. Our results provide important new insights into the regulatory mechanisms of bacterial wilt resistance in eggplant.

NAC family proteins are plant-specific transcription factors that collectively elicit a range of biological functions, are widely distributed across terrestrial plant genomes, and are considered to be one of the largest families of transcription factors^1^. NAC family proteins have an N-terminus containing a highly conserved and specific NAC domain^2^ and were originally characterized and named for their sequence similarity to the *petuniahybrida NAM* (*NO APICAL MERISTEM*) and the *Arabidopsis thaliana ATAF*1, *ATAF*2, and *CUC*2 (*CUP-SHAPED COTYLEDON*) genes^2^. They have been shown to play a variety of roles in plant growth and development, responses to abiotic and biotic stresses, and fruit maturation and to be components of hormone signal transduction pathways^3^,^4^,^5^,^6^. For example, Zhao *et al*.^7^ reported that a *NAC*1-type transcription factor (*TaNAC*-S) from wheat (*Triticumaestivum*) suppresses leaf senescence and promotes grain yield and protein content^7^. The silencing of a peach (*Prunus persica*) NAC gene that is expressed at particularly high levels in blood-fleshed peaches caused a reduction in anthocyanin pigmentation^8^. You *et al*.^9^ characterized *BdNAC* genes from *Brachypodium distachyon* and demonstrated that their expression is influenced by abiotic stresses and phytohormones^9^, and a recent study revealed that two NAC transcription repressors (*NAC*050 and *NAC*052) control flowering time by associating with the histone demethylase JMJ14^10^.

In addition to developmental roles, NAC transcription factors are known to be involved in coordinating responses to attacks by phytopathogens, including fungi, bacteria and viruses. Sun *et al*.^11^ found that 63 rice (*Oryza sativa*) NAC genes exhibited overlapping expression patterns following exposure to a variety of biotic (infection by bacterial, fungal, and viral pathogens, or parasitic plants) and abiotic (cold, salt and drought) stresses^11^. Moreover, in a study of specific plant-pathogen interactions, the wheat *GRAB*1 and *GRAB*2 proteins were found to bind to the wheat dwarf geminivirus (*WDV*) *RepA* protein, such that the conserved N-terminal domain of the GRAB proteins activates the *RepA* protein. The overexpression of the GRAB proteins were found to inhibit *WDV* DNA replication^12^. In another report, the two pathogen-responsive rice NAC transcription factors *ONAC*122 and *ONAC*131 were described as being localized in the nucleus, to exhibit transcriptional activation activity^13^, and to be expressed after *Magnaporthe grisea* infection. In another example, silencing of *ONAC*122 or *ONAC*131 expression in transgenic rice lines resulted in their increased susceptibility to *M. grisea*. These results further support an important role for *ONAC*122 and *ONAC*131 in rice disease resistance responses, suggesting their regulation of the expression of defense- and signaling-related genes^13^. Xia *et al*.^14^ showed that the expression of the wheat *TaNAC*8 gene increased following infection by the stripe rust pathogen *Puccinia striiformis f. sp. Tritici*^14^. In another study, the binding of a turnip crinkle virus coat protein to the *A. thaliana* NAC transcription factor TIP resulted in reduced viral invasion^15^. Other examples linking NAC transcription factors to disease resistance processes include reports that the *A. thaliana NAC*083 protein interacts with the *mungbean yellow mosaic India virus* (MYMIV) Rep protein^16^ and that suppression of the bread wheat *TaNAC*1 gene enhances resistance to stripe rust^17^.

When pathogenic bacteria infect plants, they generally activate the expression of a large number of genes as part of a coordinated defense response that involves the salicylic acid (SA), jasmonic acid (JA), and ethylene (ET) hormone signaling pathways. Bu *et al*.^8^ reported that *ANAC*019 and *ANAC*055 are involved in JA-mediated defense responses in *A. thaliana* and that they might regulate the transcription of the JA induced *VEGETATIVE STORAGE PROTEIN1* (*VSP*1) and *LIPOXYGENASE2* (*LOX*2) defense genes. The expression of *VSP1* and *LOX2* was also enhanced after *ANAC*019 and *ANAC*055 were overexpressed, and the double mutant (*ANAC*019 and *ANAC*055) exhibited reduced *VSP*1 and *LOX*2 expression and resistance to *Botrytis cinerea*^18^.

Bacterial Wilt (BW), which is caused by *Ralstonia solanacearum*, is considered one of the most destructive bacterial plant diseases and is known for extreme aggressiveness, with worldwide geographic distribution and a broad host range (more than 200 plant species). The dominant species of *Ralstonia solanacearum* affecting eggplant in our country is biovar III and biovar IV, which belong to race 1. Several eggplant studies investing BW include the discussion of host resistance genetic regulation^19^,^20^,^21^,^22^; pathogen separation^23^, collection, and identification of disease resistance resources^24^, and screens of resistant gene-relevant molecular markers^21^,^22^,^25^. The most documented example is the acetyl transferase *popP*2^26^, recently renamed *ripP*2^27^, which interacts with the *A. thaliana* gene *RRS1-R*^28^,^29^ (that belongs to the salicylic acid regulation pathway) and the cysteine protease RD19^30^, triggering plant immunity, making it the first avirulence gene described in *R. solanacearum*^31^. Delaspre *et al*.^32^ suggested that the bacterial wilt gene *HrpB* has an important function in infecting the host^32^. In addition, Milling *et al*.^33^ found that bacterial wilt gene *EPS* facilitates avirulence and is related to tomato bacterial wilt resistance^33^. Pensec *et al*.^31^ used an original association genetic approach combining resistant eggplant, pepper, and tomato DNA microarray and pathogenicity data to identify type-III effector (T3E) repertoires associated with virulence of the bacterial wilt pathogen *Ralstonia solanacearum* on Solanaceous crops^31^.

In this study, we describe the identification of a NAC transcription factor (*SmNAC*) from eggplant (*Solanum melongena*) based on RNA-seq data, and present data supporting its role in resistance to bacterial wilt (BW), a complex and devastating soil-borne vascular disease in plants caused by *Ralstonia solanacearum*. Bacterial wilt occurs in many crop species, including eggplant, tomato, potato, pepper, banana, ginger, cowpea, peanut, papaya, cashew, and olive plant^34^. Reports on mechanisms of host resistance are limited, and there are no reports of BW resistance regulation by NAC transcription factors. Although transcription factors containing the NAC domain have been identified in many plant species, such as in rice (*Oryza.sativa*)^35^,^36^, *A. thaliana*^37^, poplar (*Populus trichocarpa*)^37^, soybean (*Glycine max*), tobacco (*Nicotiana tabacum*)^38^, barley (*Hordeum vulgare*)^38^, and potato (*Solanum tuberosum*)^39^, to date, no NAC domain-containing proteins in eggplant have been described. Here, we present results indicating that *SmNAC* is involved in the resistance of eggplant to BW and describe its association with hormone signaling.

## Results

### Identification of *SmNAC* from *S. melongena* based on RNA-Seq data

Analysis of the RNA-seq data revealed 1,137 genes that were expressed at higher levels, and 9,048 genes that were expressed at lower levels in A0 than in A1 (Fig. S1a,b). A total of 6,087 genes were expressed at higher levels, and 5,832 were expressed at lower levels in B0 than B1, whereas 738 and 217 were expressed at higher or lower levels, respectively, in B0 compared to A0. Furthermore, 4,712 genes were expressed at higher levels and 12,523 were expressed at lower levels in B1 compared to A1. For a general characterization of the sterm tissue assemblies, an overview of the number of slimmed GO-terms for biological process is showed in Fig. S1c. The complete list of assigned GO terms for each assembly, including different levels of biological process, molecular function and cellular component, is available in it. One significantly differentially expressed gene, *SmNAC* (Gen accession: KM435267), was expressed at higher levels in BW-susceptible plants after inoculation with the pathogen (B1 plants) but at lower levels in BW-resistant plants after pathogen induction (A1 plants) (Fig. S2a). And the phenotypic symptoms of eggplants after inoculation with *Ralstonia solanacearum* were exhibited in Fig. S2b.

**Figure 1:**
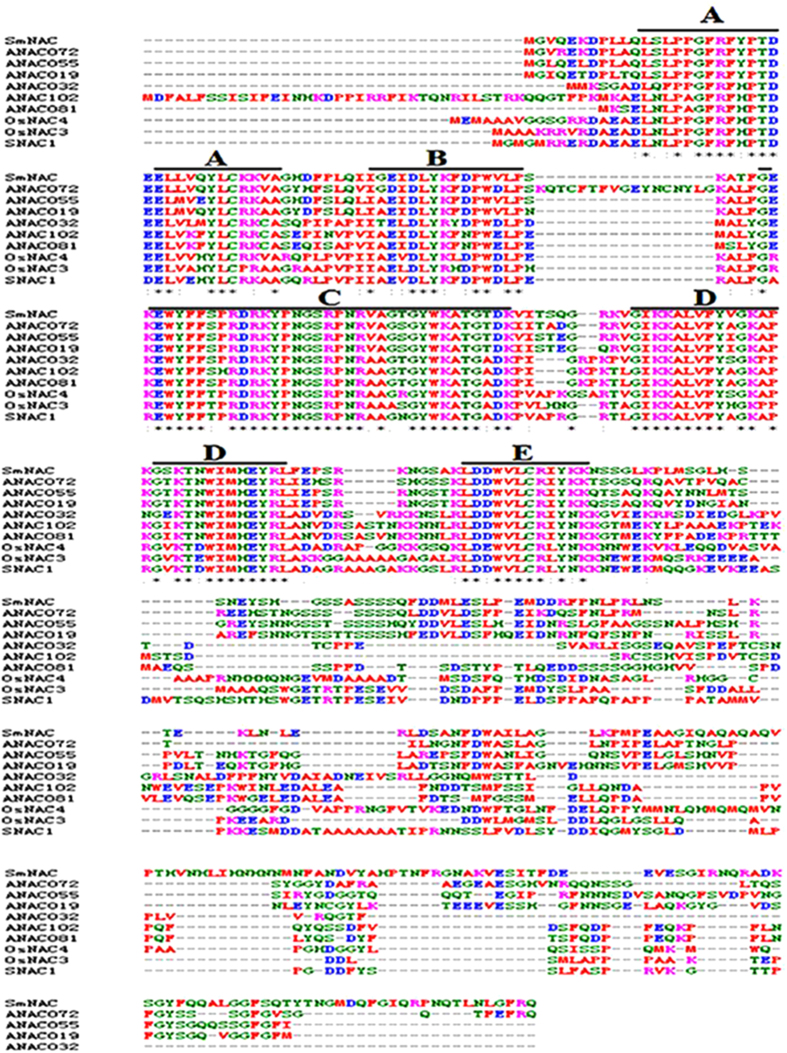
Alignment analysis of *SmNAC*. Alignment analysis of the *SmNAC* protein sequence with other plant NAC proteins. *SmNAC* was aligned with *A. thaliana ANAC*019 (AT1G52890.1), *ANAC*032 (AT1G77450.1), *ANAC*055 (AT3G15500.1), *ANAC*072 (AT4G27410.2), *ANAC*081 (AT5G08790.1) and *ANAC*102 (AT5G63790.1), and rice *OsNAC*4 (LOC_Os01g60020.1), *OsNAC*3 (LOC_Os07g12340), and *SNAC*1 (LOC_Os03g60080). The five highly conserved amino acid motifs (A–E) are indicated by black lines. Alignments were carried out using CLUSTALW v1.83 and the GeneDoc software.

Sequence analysis showed that *SmNAC* was 1,708 bp long with an ORF of 1,038 bp. The *SmNAC* protein is predicted to contain 345 amino acids and to exhibt a molecular weight of 39,035 Da and an isoelectric point of 8.94. The 5′-untranslated region (UTR) 3′-UTR are predicted to be 159 and 511 bp, respectively. The full-length predicted amino acid sequence of *SmNAC* is annotated as containing a conserved NAM domain within the N-terminal region, similar to that found in NAC proteins from other plant species (Fig. 1, Fig. S3). The *SmNAC* amino acid sequence exhibits high similarity to *ANAC*019 (AT1G52890.1), *ANAC*055 (AT3G15500.1) and *ANAC*072 (AT4G27410.2) of *A. thaliana*, and *SNAC*1 (LOC_Os03g60080), *OsNAC*3 (LOC_Os07g12340) and *OsNAC*4 (LOC_Os01g60020) of rice. However, the gene and protein structures of *SmNAC* are same in the both inbred lines (E-31 and E-32).

### Characterization of *SmNAC* expression in *S. melongena*

**Figure 2:**
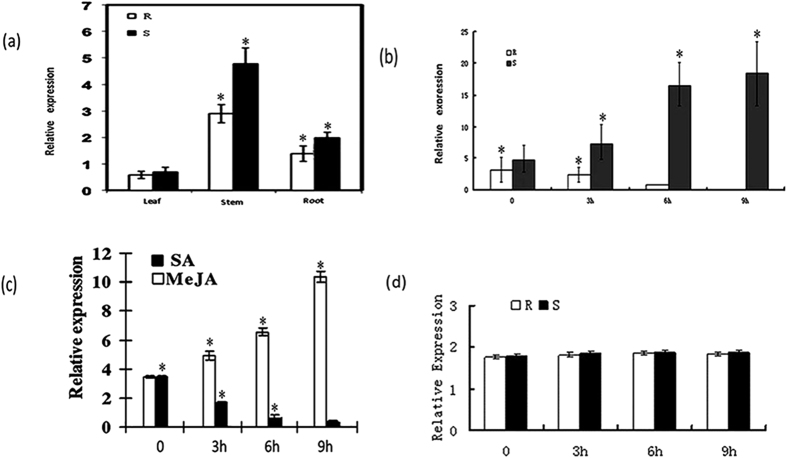
Evaluation of *SmNAC* expression in resistant and susceptible plants. The expression of *SmNAC* in resistant and susceptible plants under normal conditions (**a**), in the stems of resistant and susceptible plants after inoculation with *Ralstonia solanacearum* (**b**), and in E-32 plants after treatment with MeJA (1.5 mM) and SA (0.2 mM) (**c**), and Mock (**d**). R: resistant plants (E-31); S: susceptible plants (E-32).

To assess whether *SmNAC* is involved in the regulation of BW resistance in *S. melongena*, its expression was analyzed in inoculated resistant (E-31) and susceptible (E-32) plants (Fig. 2). Under uninfected conditions, *SmNAC* expression was detected in roots, stems and leaves, with the highest expression in stems and the lowest expression in leaves (Fig. 2a). Prior to inoculation, *SmNAC* expression was higher in susceptible (E-32) than in resistant (E-31) plants. After inoculation with *R. solanacearum* strains, *SmNAC* transcript levels increased in susceptible *S. melongena* (E-32) but decreased in resistant *S. melongena* (E-31) plants, whereas they were no longer detected at 9 h after inoculation. However, the expression was substantially greater in E-32 plants (Fig. 2b). At the same time, E-32 plants weretreated with MeJA and JA strains, and the results indicate that MeJA, but not SA, can induce the expression of *SmNAC*. Taken together, these results suggest that *SmNAC* might play a role in the regulation of BW-resistance in *S. melongena*.

### Immunohistochemical localization of *SmNAC*

**Figure 3:**
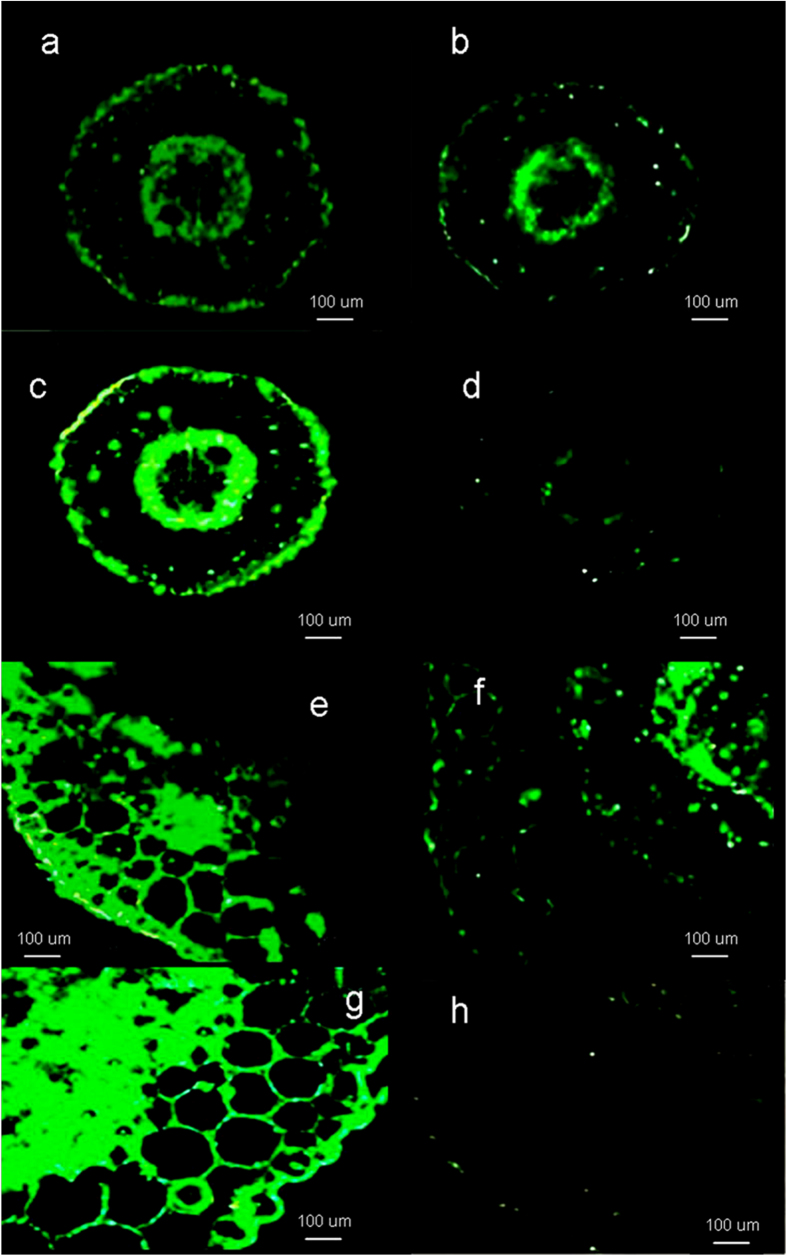
Immunohistochemical localization analysis of *SmNAC*. Fresh ‘E-32’ *S. melongena* stem and root tissues were used, and cross-sections of eggplant tissue labeled with purified *SmNAC* antibodies and visualized using an anti-rabbit IgG conjugated to green fluorescent protein. Scale bars, 100 μm. Each experiment repeated three times. (**a**) *SmNAC* protein distribution in the root of an uninfected susceptible plant (E-32). (**b**) *SmNAC* protein distribution in the root of an uninfected resistant plant (E-31). (**c**) *SmNAC* protein distribution in the root of a susceptible plant (E-32) after inoculation with *Ralstonia solanacearum* for 6 h. (**d**) *SmNAC* protein distribution in the root of a resistant plant (E-31) after inoculation with *R. solanacearum* for 6 h. (**e**) *SmNAC* protein distribution in the stem of an uninfected susceptible plant (E-32). (**f**) *SmNAC* protein distribution in the stem of an uninfected resistant plant (E-31). (**g**) *SmNAC* protein distribution in the stem of a susceptible plant (E-32) after inoculation with *R. solanacearum* for 6 h. (**h**) *SmNAC* protein distribution in the stem of a resistant plant (E-31) after inoculation with *R. solanacearum* for 6 h.

In order to analyze the tissue specific localization of *SmNAC* in *S. melongena*, tissue sections of stems and roots were treated with antibodies coupled to a green fluorescent dye. In the root sections, two zones of strong *SmNAC* antibody staining corresponding to the phloem and xylem were observed (Fig. 3a,b). After inoculation with BW, the staining was stronger in the roots of E-32 plants than in those of E-31 plants (Fig. 3c,d), and the same pattern was observed in stem tissues (Fig. 3e–h). Because the signal intensity increased in E-32 but decreased in E-31 plants, we concluded that *R. solanacearum* can induce the accumulation of *SmNAC* in susceptible but not resistant *S. melongena* plants.

### Subcellular localization and transcriptional activation of *SmNAC*

**Figure 4:**
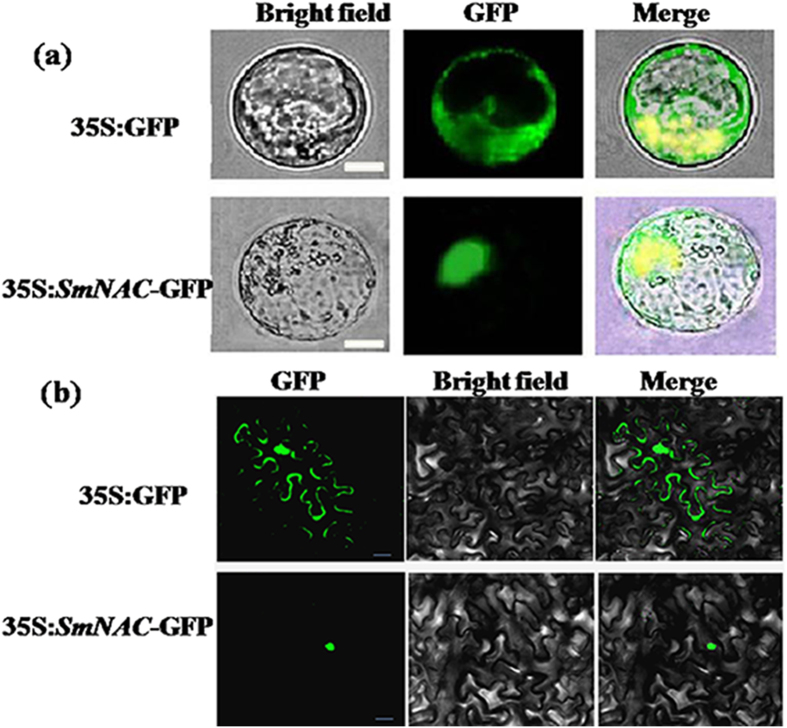
Subcellular localization of *SmNAC*. The PCR products were sub-cloned into the pEZS-NL-GFP vector in-frame with the green fluorescent protein (GFP) sequence, resulting in *SmNAC*-GFP vectors under the control of the CaMV 35S promoter. The fusion construct vector and the control GFP alone vector were introduced into *S. melongena* (E-32) protoplasts. GFP fluorescence was visualized using a laser scanning confocal microscope. All transient expression assays were repeated at least three times. (**a**) Eggplant (E-32) protoplasts were transiently transformed with *SmNAC*-GFP (green fluorescent protein) or GFP expressing constructs and fluorescence was visualized using a fluorescence microscope. Images were acquired in either dark field (green fluorescence) or bright field modes. Scale bars = 25 μm. (**b**) Ben’s tobacco protoplasts were transiently transformed with *SmNAC*-GFP (green fluorescent protein) or GFP expressing constructs and fluorescence was visualized using a fluorescence microscope. Images were acquired in either dark field (green fluorescence) or bright field modes. Scale bars = 25 μm.

To establish the subcellular localization of *SmNAC*, a *SmNAC* and GFP fusion protein under the control of a 35S promoter was transiently expressed in *S. melongena* (E-32) and Ben’s tobacco protoplasts. Fluorescence signal localized to the nuclei (Fig. 4a,b). In contrast, control cells expressing GFP alone exhibited an even distribution of the GFP signal in both the cytoplasm and the nuclei.

**Figure 5:**
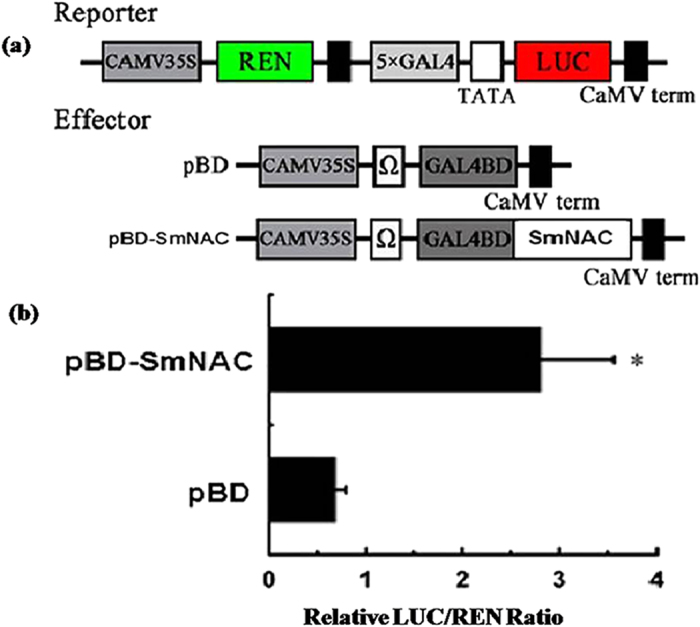
Transcriptional activation of *SmNAC*. The *SmNAC* coding sequence without the stop codon was cloned into the reconstructed GAL4-DBD vector. To assay the binding activity of *SmNAC* to the *ICS*1 promoter, the *ICS*1 promoter was cloned into a pGreenII 0800-LUC double reporter vector, whereas *SmNAC* was inserted into the pGreenII 62-SK vector, generating the effector construct. The effector and reporter plasmids were co-transformed into *S. melongena* protoplasts as previously described and incubated as described above. LUC and REN luciferase activities were measured using a dual luciferase assay kit (Promega, USA). The analysis was performed using the Luminoskan Ascent Microplate Luminometer (Thermo, USA) with a 5 s delay and 15 s integration time. The binding activity of *SmNAC* to the *ICS1* promoter was measured as a ratio of LUC to REN. At least six transient assays were measured for each assay. (**a**) The dual luciferase reporter construct contains a LUC reporter gene driven by the 35S (TATA box) promoter with five GAL4-binding elements, whereas each of the effectors contain a GAL4 DNA-binding domain (GAL4-BD); pBD was used as a negative control. *SmNAC* was linked to the GAL4-BD sequence and expression was driven by a 35S promoter. (**b**) Transactivation activity of *SmNAC*. Plasmid combinations of the dual REN/ LUC reporter, and effectors were co-transformed into eggplant protoplasts. After 12 h, the transactivation activity of *SmNAC* was measured as a ratio of LUC to REN. Each value represents the means of three biological replicates, and vertical bars represent the S.E. The asterisk indicates a significant difference at the 5% level.

The transcriptional activation of *SmNAC* was assessed in a transient expression assay using E-32 protoplasts. Full-length *SmNAC* fused to the GAL4 DNA-binding domain (GAL4-BD) was used as the effector. A dual luciferase reporter vector containing five copies of the GAL4 DNA-binding element and a minimal TATA region of a 35S promoter was fused to the firefly luciferase (LUC) reporter together witha Renilla luciferase (REN) reporter controlled by a 35S promoter. Activation of these reporters was used as an internal control for successful transformation (Fig. 5a), and an empty GAL4-BD (pBD) vector was used as a negative control. Compared with the negative control (pBD), the presence of *SmNAC* strongly increased the expression of the LUC reporter gene (Fig. 5b), and the LUC/REN ratio of *SmNAC* was significantly higher than that of the negative control.

### Overexpression of *SmNAC* in *S. melongena* reduces resistance to BW

**Figure 6:**
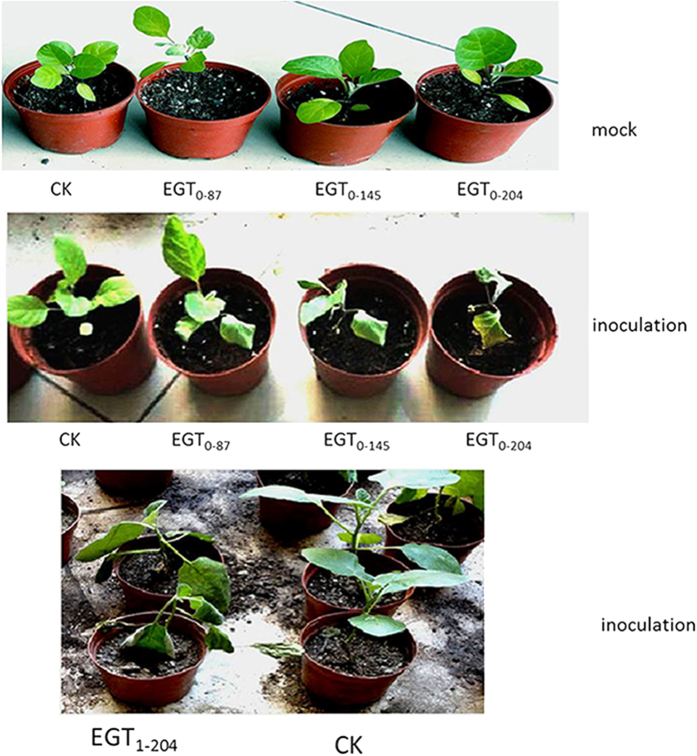
Identification of bacterial wilt (BW) resistance in *SmNAC* overexpressing eggplant lines. CK represents non-transgenic E-31 plants; EGT_0–87_, EGT_0–145_, EGT_0–204_ are overexpressing *SmNAC* transgenic lines T_0_); and EGT_1–204_ indicates the T_1_ transgenic plant of EGT_0–204_. BW resistance was measured 15 d after pathogen inoculation. Whereas the non-transgenic resistant plant E-31 exhibited no wilt symptoms, the T_0_ and T_1_ overexpressing *SmNAC* plants exhibited clear wilt symptoms.

In order to analyze the tissue specific localization of *SmNAC* in *SmNAC*-overexpressing plants and *SmNAC*-RNAi transgenic plants, tissue sections of stems were treated with antibodies coupled to a green fluorescent dye. Two zones of strong *SmNAC* antibody staining corresponding to the phloem and xylem were observed (Fig. S6a,c). After overexpressing *SmNAC*, the staining was stronger in the stem of a *SmNAC*-overexpressing plant (EGT_1–87_) than in those of a non-transgenic plant (E-31) (Fig. S6a,b); After knocking-down *SmNAC*, the staining was weaken in the stem of the *SmNAC*-RNAi transgenic plant (RNAi-2-1) than in those of a non-transgenic plant (E-32) (Fig. S6c,d). The potential function of *SmNAC* in disease resistance was evaluated using transgenic eggplant plant lines overexpressing *SmNAC* and RNAi-*SmNAC* in which *SmNAC* expression was suppressed. We generated a thousand hypocotyl explants of the *SmNAC*-overexpressing lines, in which transgene expression was driven by a constitutive CaMV 35S promoter; six transgenic *S. melongena* plants were obtained (EGT_0–5_, EGT_0–23_, EGT_0–43_, EGT_0–87_, EGT_0–145_ and EGT_0–204_). The presence of the transgene was verified by genomic Southern blot, qRT-PCR and Western blot analyses (Fig. S4a–c). *SmNAC* expression levels were higher in all of the transgenic plants but not in the non-transgenic plants (E-31) (Fig. S4b), the levels of *SmNAC* protein in all transgenic plants exhibited the same changes (Fig. S4c). Three T_1_ transgenic lines (EGT_1–87_, EGT_1–145_ and EGT_1–204_) were obtained by selfing the primary transformants (T_0_) and these were used to assess BW resistance. Resistance was reduced in the T_0_ and T_1_ transgenic *SmNAC* overexpressing plants (Table 1, Fig. 6) and the non-transgenic plants (E-31) did not exhibit any symptoms. We conclude from these results that the overexpression of *SmNAC* in *S. melongena* reduces resistance to BW.

**Figure 7:**
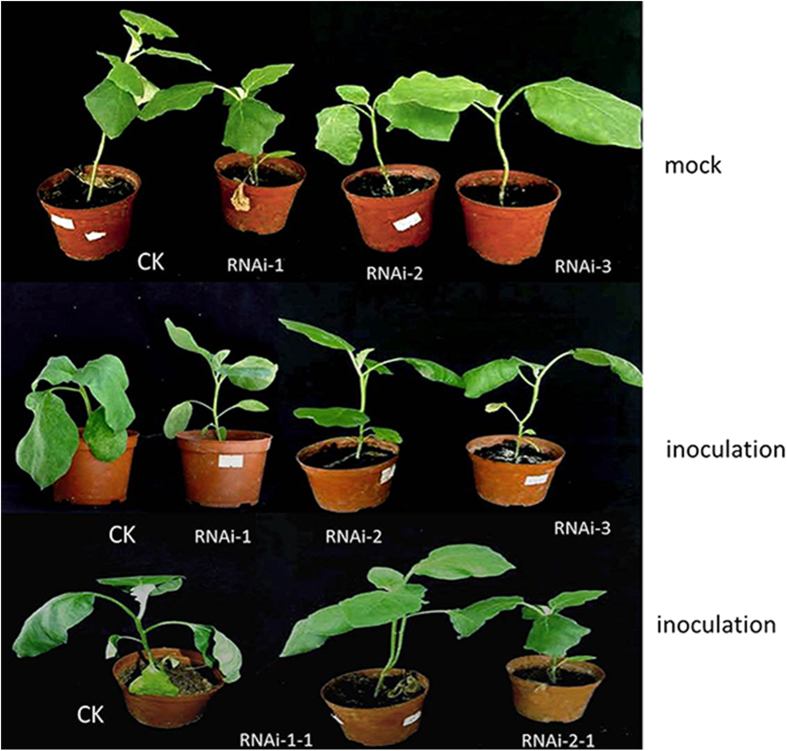
Identification of bacterial wilt (BW) resistance in RNAi-*SmNAC* transgenic eggplants. CK represents non-transgenic E-32 plants; RNAi-1, -2, and -3 represents RNAi-*SmNAC* transgenic eggplants (T_0_); RNAi-1-1 and RNAi-2-1 are T_1_ progeny. The BW resistance of plants was assessed at 15 days after pathogen inoculation. The non-transgenic susceptible plants from the E-32 line exhibited wilt symptoms, whereas the T_0_ and T_1_ transgenic RNAi-*SmNAC* plants did not.

The association that we observed was further supported by our evaluation of the effects of *SmNAC* silencing in susceptible plants (E-32) using RNAi. We again used 1,000 hypocotyls as explants for transformation, and obtained 5 RNAi-*SmNAC* transgenic plants, which were screened for the presence of the transgene by Southern blot, qRT-PCR and Western blot analysis (Fig. S5a–c). *SmNAC* transcript levels were reduced in the *SmNAC*-RNAi transgenic (T_0_) plants compared to the non-transgenic plants (E-32), and no hybridization signals of *SmNAC* protein in any of the RNAi-treated plants were detected (Fig. S5c). The T_0_ and T_1_ transgenic plants were BW resistant after silencing of *SmNAC* (Table 2, Fig. 7). At 7 days after bacterial inoculation, the non-transgenic plants exhibited BW symptoms, whereas the RNAi plants did not; at 13 days after inoculation, more than half of the non-transgenic plants exhibited BW symptoms, and the *SmNAC*-RNAi plants also began to develop leaf wilt. We conclude that the silencing of *SmNAC* in susceptible *S. melongena* delays the presentation of BW symptoms, thereby increasing resistance to BW.

**Figure 8:**
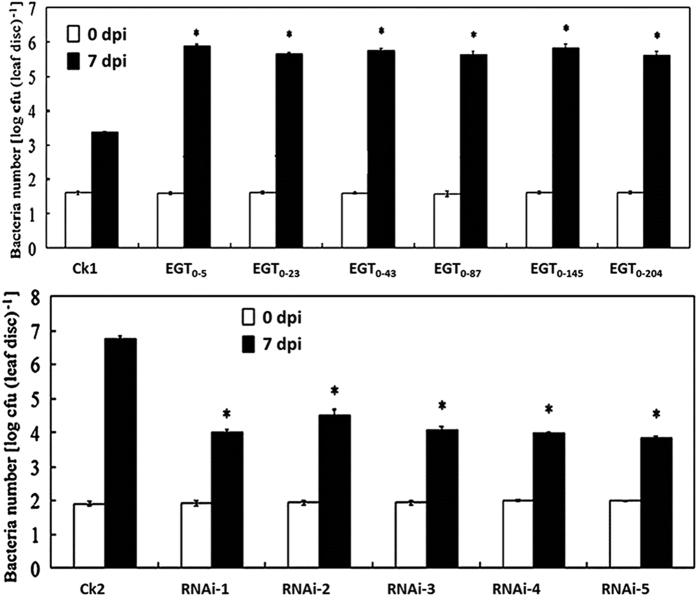
Measurement of bacterial growth in transgenic eggplants. Briefly, using the syringe inoculation method, bacteria wilt was scraped off a fresh plate, resuspended in sterile water to 10^5^ colony-forming units (c.f.u.) ml^−1^, and pressure-infiltrated into leaves with a needleless syringe. After 7 days, leaves were harvested and surface sterilized (30 s in 70% ethanol, followed by 30 s in sterile distilled water) for the spray inoculation method. Leaf discs from different leaves were ground in 10 mM MgCl_2_ using a microfuge tube glass pestle. After homogenization, the samples were thoroughly vortex-mixed and diluted 1:10 serially. Samples were finally plated on TZC solid medium (3 g casein hydrolysate, 5 g peptone, and 10 g glucose (pH 7.0). The plates were incubated at 28 °C for 2 days, after which the colony-forming units were counted. CK1 represents non-transgenic plants from the E-31 line; EGT_0–87_, EGT_0–145_, and EGT_0–204_ are the *SmNAC* overexpressing transgenic eggplants (T_0_). CK2 represents non-transgenic plants from the E-32 line; RNAi-1, -2, -3, -4, and -5 are RNAi-*SmNAC* transgenic plants (T_0_). Error bars represent standard error, and the experiments were repeated at least three times with similar results. Asterisks indicate a significant difference at P < 0.05 compared with the non-transgenic plants.

Quantification of bacteria in the pathogen-infected eggplant leaves revealed that the *SmNAC*-overexpressing plants exhibited a significantly increased bacterial population compared to the non-transgenic plants (E-31) at 7 dpi, whereas the leaves of the *SmNAC*-RNAi plants exhibited fewer bacteria than those of the non-transgenic plants (E-32) (Fig. 8).

### The effect of *SmNAC* on the expression of genes involved in defense signaling pathways

**Figure 9:**
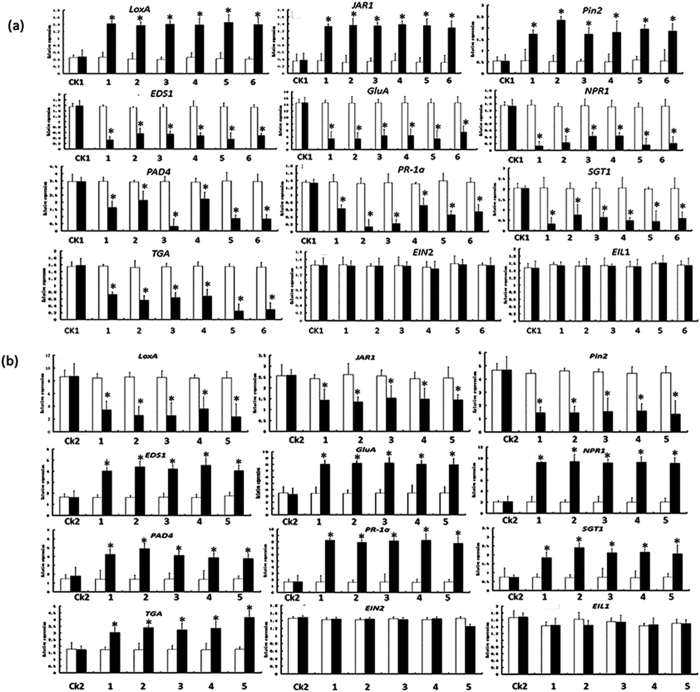
qRT-PCR analysis of the expression of defense signaling genes in transgenic eggplants. Quantitative reverse-transcription PCR (qPCR) was performed using a SYBR Premix Ex Taq kit (TaKaRa, Dalian, China), following the manufacturer’s protocols. Triplicate qPCR reactions were performed for each sample and the relative gene expression data was analyzed using the 2^−ΔΔ^Ct method. (**a**) qRT-PCR analysis of defense signaling genes in *SmNAC* oveexpressing plants. CK represents non-transgenic plants from the E-31 line, whereas 1–3 show the *SmNAC* overexpressing transgenic T_0_ plants EGT_0–87_, EGT_0–145_, and EGT_0–204_, and 4–6 show the *SmNAC* overexpressing transgenic T_1_ plants EGT_1–87_, EGT_1–145_, and EGT_1–204_. (**b**) qRT-PCR analysis of defense signaling genes in RNAi-*SmNAC* plants. CK represents non-transgenic plants from line E-32. 1–5 show the RNAi-*SmNAC* transgenic plants (T0) RNAi-1, RNAi-2, RNAi-3, RNAi-4, and RNAi-5.

We analyzed the effect of *SmNAC* on the expression of genes involved in the JA and SA defense signaling pathways. Transcript levels were quantified by qRT-PCR, and it was found that the expression of genes in the JA signaling pathway (*JAR*1, *Pin2*, *LoxA*) was higher in the *SmNAC* overexpressing lines (Fig. 9a) and lower in the *SmNAC*-RNAi lines (Fig. 9b) compared with control plants. In contrast, the expression of genes in the SA signaling pathway (*EDS1, GluA, NPR1, TGA, SGT1, PAD4*, *PR-*1a) was significantly lower in the *SmNAC* overexpressing lines (Fig. 9a) and higher in the RNAi-silenced *SmNAC* lines (Fig. 9b). These results indicate that *SmNAC* promotes and represses the transcription of genes in the JA and SA pathways, respectively.

### *SmNAC* regulates SA biosynthetic genes

We next measured the effects of *R. solanacearum* infection on SA and JA levels in the *SmNAC* overexpressing plants, *SmNAC*-RNAi plants, and non-transgenic plants (E-31, E-32). The SA concentrations were increased in the non-transgenic resistant plants (E-31, CK_1_) compared to the *SmNAC* overexpressing plants (Table 3), and they were significantly increased in the *SmNAC*-RNAi plants compared to the non-transgenic susceptible plants (E-32, CK_2_). In addition, we observed that the JA content of all the susceptible plants differed significantly from that of the resistant plants after inoculation with the pathogen (Table 4). Based on these findings, we conclude that the *SmNAC* gene represses SA biosynthesis in *S. melongena* and might directly mediate genetic control over SA biosynthesis or metabolism.

**Figure 10:**
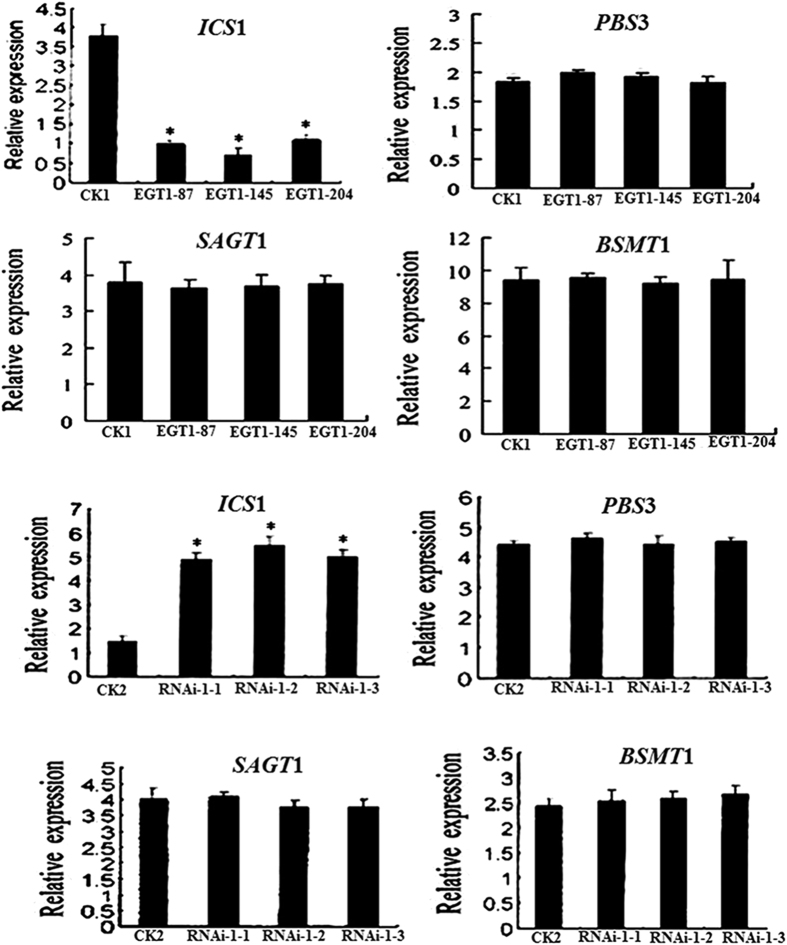
Expression of salicylic acid biosynthetic and catabolic genes in both *SmNAC* overexpressing plants and RNAi-*SmNAC* plants. EGT_1–87_, EGT_1–145_, and EGT_1–204_ are the T_1_ generation of *SmNAC* overexpressing plants from the T_0_ generation plants, EGT_0–87_, EGT_0–145_, and EGT_0–204_. CK1 shows E-31. RNAi-1-1, RNAi-2-1, and RNAi-3-1 are the T_1_ generation RNAi-*SmNAC* plants from the T_0_ generation plants, RNAi-1, RNAi-2, and RNAi-3. CK2 shows E-32.

To determine whether SA-biosynthetic or SA-catabolic genes are regulated by *SmNAC*, we measured the expression levels of four associated genes (*ICS*1, *PBS*3*, SAGT*1, *BSMT*1). *ICS*1 expression was increased in the non-transgenic resistant plants (E-31) compared to the *SmNAC* overexpressing or non-transgenic susceptible plants (E-32), whereas the expression of the other three genes (*PBS3, SAGT1*, *BSMT1*) was not significantly different between the non-transgenic resistant plants (E-31) and the *SmNAC* overexpressing lines. Similarly, we found that *ICS*1 expression levels were higher in the *SmNAC*-RNAi lines than in the non-transgenic susceptible plants (E-32), but that there were no significant differences in the expression levels of the other three SA-related genes. This suggests that the three SA catabolic genes are not affected by *SmNAC* (Fig. 10), but that the SA biosynthetic gene *ICS*1 is regulated by *SmNAC* expression.

### *SmNAC* regulates *ICS*1 through direct interaction with its promoter

A 1,697 bp region of the predicted *ICS*1 promoter, which we identified by genome walking PCR, was analyzed using the PLACE and Plant-CARE databases (Table S3). Based on these analyses, we found six NAC core-binding sites (CACG). The promoter sequence also contained the core *cis*-acting elements TATA and CAAT, several light response *cis*-acting elements, heat stress response elements, an elicitor-responsive element, and a *cis*-acting element involved in defense and stress responses.

**Figure 11:**
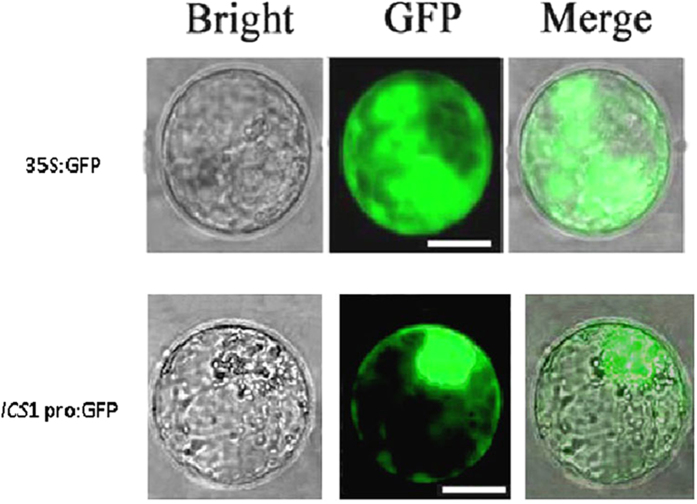
Detection of *ICS1* promoter activity in eggplant (E-32) protoplasts. GFP reporters driven by the *ICS1* promoter (*ICS1 pro*::GFP) and a CaMV 35S promoter (35S::GFP, positive control) were transiently expressed in eggplant E-32 protoplasts. After incubation for 12 h, GFP fluorescence was visualized by fluorescence microscopy. Scale bar = 25 μm. The experiment was repeated three times with similar results.

**Figure 12:**
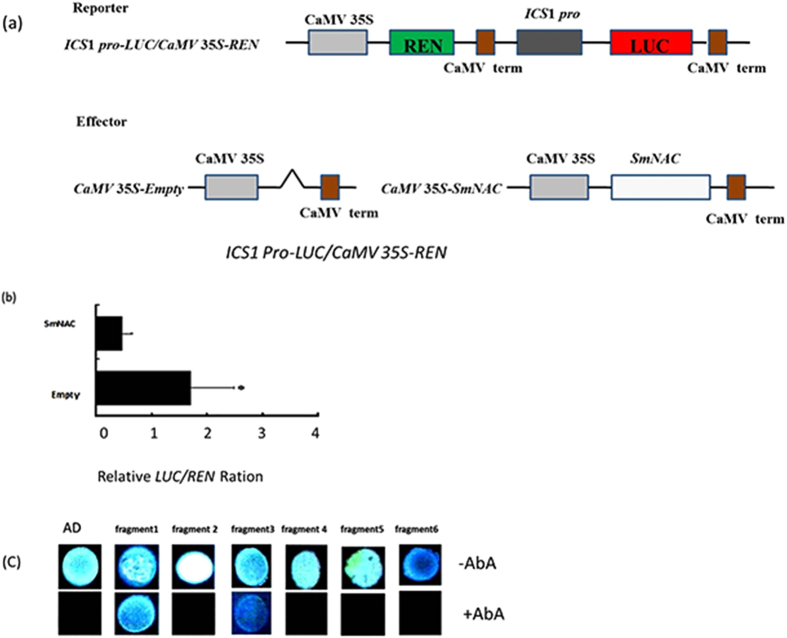
*SmNAC* represses the *ICS*1 promoter in a dual luciferase assay and yeast-one hybrid assay. (**a**) A schematic representation of the double reporter and effector plasmids used. The double-reporter plasmid contained the *ICS*1 promoter fused to sequences encoding the LUC luciferase and the REN luciferase driven by the *CaMV*35S promoter. The effector plasmid contained *SmNAC* driven by a *CaMV*35S promoter. (**b**) *SmNAC* represses the *ICS*1 promoter. The reporter and effector vectors, as indicated, were co-transformed into eggplant E-32 protoplasts, which were incubated for 12 h. The activation of the *ICS*1 promoter by *SmNAC* was based on the ratio of LUC to REN. The asterisk indicates a significant difference at the 5% of the level compared to the empty effector vector. Each value represents the means of three biological replicates. (**c**) Yeast-1-hybrid (Y1H) assay of *SmNAC* and *ICS*1 promoter fragments. Fragment 1: −1 to−370 bp; fragment 2: −380 to −520 bp; fragment 3: −550 to −750 bp; fragment 4: −952 to −1170 bp; fragment 5: −1180 to −1318 bp; fragment 6: −1410 to −1570 bp. +AbA = SD/−Leu/+AbA medium, including the addition of 100 μm AbA. −AbA = SD/−Leu medium, no AbA added.

To monitor *ICS*1 promoter activation, an *ICS*1 *pro-*GFP vector and a positive control vector (35 S::GFP) were transformed into *S. melongena* protoplasts, and due to the fluorescent signal observed in both types of transformed protoplasts (Fig. 11), we conclude that the *ICS*1 promoter was sufficient to drive GFP expression. To investigate whether *SmNAC* could directly activate the *ICS*1 promoter in plant cells, a dual luciferase-based transactivation assay was performed using *S. melongena* E-32 protoplasts. The dual luciferase reporter plasmid harbored the *ICS*1 promoter fused to *LUC* and *REN* that are driven by the CaMV35S promoter (CaMV35S-*REN*/*ICS1pro*-LUC). An effector plasmid harboring *SmNAC* was expressed under the control of the CaMV 35S promoter (Fig. 12a). We observed that when an *ICS*1 *pro*-*LUC* reporter construct was co-transformed with the effector, CaMV 35S*-SmNAC*, the *LUC*/*REN* ratio was significantly reduced compared to the control ratio (Fig. 12b). Similarly, yeast one hybrid (Y1H) (Fig. 12c) analyses indicated that *SmNAC* binds to the *ICS*1 promoter at two binding sites, from −1 to −370 and from −550 to −750. We conclude that *SmNAC* functions as a transcriptional repressor that regulates *ICS*1 expression by directly binding to its promoter and repressing the transcription of *ICS*1.

### The effects of exogenous SA on BW resistance in *SmNAC* overexpressing plants

**Figure 13:**
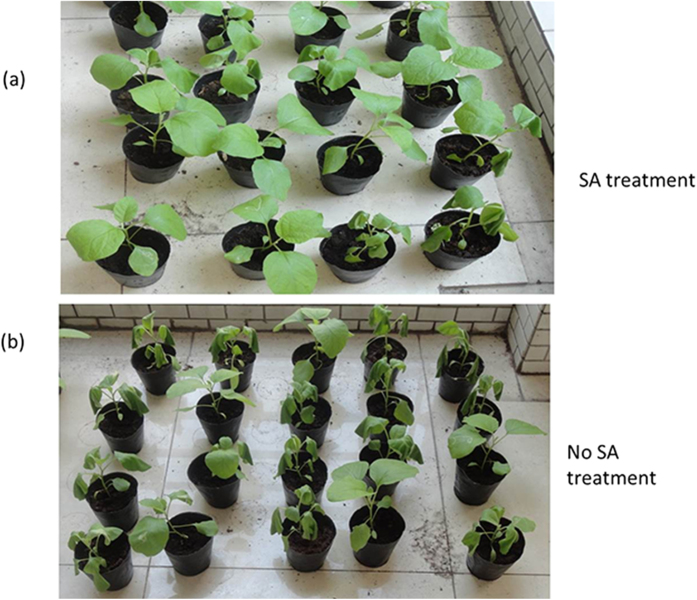
Effect of exogenous salicylic acid on bacterial wilt (BW) resistance in *SmNAC* overexpressing plants. Transgenic EGT_1–204_ plants were sprayed with 0.2 mM salicylic acid and water prior to inoculation with the pathogen. After 10 d, the plants were assessed for bacterial wilt resistance.

To examine the effects of exogenous SA on BW resistance in *S. melongena*, 60 *SmNAC* overexpressing seedlings (EGT_1–204_) and susceptible *S. melongena* (E-32) seedlings were sprayed with 0.2 mM SA prior to infection with *R. solanacearum*. At 7 days after inoculation, 38% of the EGT_1–204_ and 55% of the E-32 plants that had been treated with SA began to wilt, whereas the *SmNAC* overexpressing (EGT_1–204_) and susceptible *S. melongena* (E-32) plants treated with SA did not exhibit any BW symptoms. At 10 days after infection, 18% of the EGT_1–204_ plants and 27% of the E-32 plants treated with SA began to wilt, whereas at 15 days after inoculation, all of the non-treated EGT_1–204_ and E-32 plants presented with significant wilt symptoms. However, all of the plants treated with SA developed wilt at 20 days after inoculation (Table 5). These results suggest that SA affects BW resistance in *S. melongena* and, further, that reducing SA levels reduces resistance to BW (Fig. 13).

## Discussion

NAC transcription factors elicit a variety of biological functions in plant development, including the regulation of responses to abiotic or biotic stresses, and they can be broadly classified into two large groups, I and II, based on structural features^35^. A series of studies have indicated that they are also involved in the regulation of the plant defense networks in response to attacks by a wide range of microbial pathogens and insects. For example, *A. thaliana ATAF*2 is thought to act as a repressor of pathogenesis-related (PR) gene expression^40^, whereas *ATAF*1 suppresses defense responses following pathogen infection^41^. *OsNAC*3 was found to be involved in responses to abiotic stress^42^, rice *OsNAC*6 and *OsNAC*19 are thought to promote resistance to the rice blast fungus^43^, and NAC genes have been found to be induced in oil seed rape after flea beetle colonization and *Sclerotinia sclerotiorum* infection^44^.

In this study, *SmNAC* from eggplant was identified from an analysis of RNA-Seq data and was shown to be most similar in sequence to the NAC proteins described above (*OsNAC*3, *OsNAC*4, *SNAC*1, *ANAC*081, *ANAC*102, *ANAC*32, *ANAC*019, *ANAC*055 and *ANAC*072) (Fig. 1). The expression levels of *SmNAC* differed between BW-resistant (E-31) and susceptible (E-32) plants (Fig. 2). This might be attributable to different promoter structures of *SmNAC* between the two plants, although future studies will be needed to confirm this. *SmNAC* expression was induced by infection with the bacterium *R. solanacearum*, the causal agent of BW. *SmNAC* overexpression in resistant *S. melongena* plants reduced BW resistance (Fig. 6), whereas silencing of *SmNAC* in susceptible *S. melongena* resulted in increased BW resistance (Fig. 7). We conclude from these results that *SmNAC* expression suppresses resistance to BW.

The specific triggers and mechanisms associated with BW resistance in *S. melongena* are currently unknown. Plant immunity-related signals, such as salicylic acid (SA), jasmonic acid (JA), and ethylene (ET) are known to play essential roles in defense against pathogens, and immune responses are coordinated through a complex signaling crosstalk among these hormone-regulated pathways. For example, JA can suppress SA-mediated defenses^45^,^46^. Several reports have shown that the ET and SA signaling pathways of tomato plants are activated during the development of BW disease resistance. Furthermore, the JA, ET, and SA defense signaling pathways have been shown to interact synergistically to increase resistance to BW in tomato plants^47^,^48^. Milling *et al*.^33^ found that the expression of key genes of the JA pathway did not enhance BW resistance in tomato^33^, and in this study, the expression of three marker genes of the JA signal pathway (*JAR1*, *PIN2*, *LOXA*) increased in *SmNAC* overexpressing transgenic lines and in susceptible non-transgenic eggplant (E-32) but decreased in the RNAi-silenced *SmNAC* transgenic plants and in the resistant non-transgenic eggplant (E-31) (Fig. 9). However, the JA content of all the susceptible plants were significantly higher than those of the resistant plants (Table 4) after pathogen inoculation. We therefore deduce that the genes of the JA signaling pathway can reduce BW resistance in eggplant. In contrast, the expression of key genes in the SA signaling pathway (*EDS*1*, GLUA, NPR*1*, TGA, SGT*1*, PAD4*, and *PR-1A*) decreased in *SmNAC* overexpressing transgenic *S. melongena* lines but increased in RNAi-silenced *SmNAC* transgenic plants (Fig. 9), and the SA levels of resistant plants were significantly higher than those of the susceptible plants (Table 3). The SA-related defense pathway has been well characterized in *A. thaliana,* and the *NPR*1 gene has been shown to be required to promote *TGA* expression and to activate downstream defense genes^47^. Our data support this same function for SA in *S. melongena.* We note that in our analyses, the expression of *EIN*2 and *EIL*1 in the ET signaling pathway exhibited no significant differences between the transgenic and non-transgenic plants, suggesting that the ET signaling pathway does not play a role in *SmNAC*-mediated regulation of BW-resistance in eggplant. The MAPK pathway has also been proposed as a defense mechanism against BW in tomato plants^47^, although the expressions of genes in this pathway were not evaluated in *S. melongena* in this or previous studies^49^. Because *SmNAC* overexpression in resistant *S. melongena* reduced BW resistance compared to non-transgenic resistant plants (E-31), *SmNAC* silencing in susceptible *S. melongena* might increase BW resistance compared to non-transgenic susceptible *S. melongena* (E-32). We conclude that the expression of *SmNAC* reduces BW resistance by suppressing gene expression in the SA signaling pathway. Kloek *et al*.^50^ reported that increasing SA levels in *A. thaliana* enhanced resistance to the bacterium *Pseudomonas syringae*^50^. In *A. thaliana*, coronatine, a toxin produced by *P. syringae*, activates the expression of *ANAC*019, *ANAC*055, and *ANAC*072 through the action of the *MYC*2 transcription factor. This results in an inhibition of SA accumulation by repressing the *ICS*1 gene and activating the *BSMT*1 gene. The end result is a decrease in resistance to the BW pathogen. In our study, *SmNAC* suppresses the activity of *ICS*1 by interacting with the *ICS*1 promoter in *S. melongena* (Fig. 12), whereas *SmNAC* does not regulate the expression of *PBS*3, *SAGT*1or *BSMT*1 (Fig. 10). The SA levels in *SmNAC* overexpressing transgenic plants were lower than those in non-transgenic plants (E-31), but SA levels in RNAi-silenced *SmNAC* transgenic plants were higher than those in non-transgenic susceptible *S. melongena* (E-32). Taken together, these results suggest that *SmNAC* represses the accumulation of SA by inhibiting *ICS*1 expression, thereby lowering BW resistance. Our discovery might be important for the development of strategies for reducing loss of eggplant crops to BW infection.

## Materials and Methods

### Plant material

We used two *Solanum melongena* inbred lines, ‘E-31’ (R) and ‘E-32’ (S), obtained from South China Agricultural University (Guangzhou, China). ‘E-31’ (R) and ‘E-32’ (S) have been described by Xiao *et al*.^49^.

### Inoculation with *R. solanacearum* strains

*S. melongena* seedlings were grown in a mixture of turf soil and perlite (2:1) in a greenhouse with natural light at 20–25 °C. At the two- or three-leaf stage, seedlings were transferred to a phytotron maintained at (30 ± 2)/(25 ± 2) °C day/night (12 h day length) and 90% relative humidity. A highly virulent *R. solanacearum* strain (race1) was isolated from the BW susceptible *S. melongena* strain (E-32). A single colony of the virulent type was grown at 30 °C for 48 h on TZC medium. The inoculum was incubated in liquid TZC medium containing 3 g casein hydrolysate, 5 g peptone, and 10 g glucose (pH 7.0) by shaking in a water bath at 30 °C for 24 h. After incubation, the density of the suspension was determined using a spectrophotometer and adjusted to 1 × 10^8^ colony forming units (cfu)/ml. Seedlings were inoculated at the four- or five-leaf stage by wounding the roots and incubating them in the bacterial suspension for 20 min. The plants were inoculated for 4 weeks. After 7 d of inoculation, susceptible plants began showing symptoms of BW.

### Total RNA extraction, construction of RNA-Seq libraries and data analysis

RNA-Seq analysis was performed on resistant (E-31) and susceptible (E-32) eggplant lines. Lines A0 and A1 corresponded to mock-inoculated and pathogen-inoculated E-31 plants at 7 days post-inoculation (dpi). Similarly, B0 and B1 correspond to mock-inoculated and pathogen-inoculated E-32 plants at also at 7 dpi. RNA was extracted from ten plants stems of each of these four lines and used for RNA-Seq library construction, with 2 replicates for each treatment. Total RNA was extracted using TRIZOL reagent (Huayueyang, Beijing, China) and then treated with RNase-free DNaseI (TaKaRa, Dalian, China), according to the manufacturer’s protocols. PolyA mRNAs were purified using oligo-dT-attached magnetic beads. One microgram of purified mRNA was cleaved into 200-500 bp fragments by super-sonication, and the cleaved mRNAs were used as templates for the RNA-Seq libraries and digital gene expression (DGE) profile analyses. First-strand cDNA was synthesized using an M-MLV RTase cDNA synthesis kit (TaKaRa, China) following the manufacturer’s protocols, prior to purification using the QiaQuick PCR extraction kit, end repair, addition of dA-tails, and ligation to Illumina adapters. The ligation products were size resolved by agarose gel electrophoresis, and the fragments were excised for PCR amplification. The amplified fragments were sequenced using an Illumina HiSeq™ 2000 by Gene Denovo Co. (Guangzhou, China).

For sequence assembly, raw reads were filtered to remove adaptor sequences, empty reads, and low-quality sequences. The resulting cleaned reads were *de novo* assembled^51^, and unigenes were annotated using the NCBI BLASTx function (http://www.ncbi.nlm.nih.gov/BLAST/) with an E-value threshold of 1E^−5^, the NCBI nr database (http://www.ncbi.nlm.nih.gov), the Swiss-Prot protein database (http://www. expasy.ch/sprot), the KEGG database (http://www.genome.jp/kegg), and the COG database (http://www.ncbi.nlm.nih.gov/COG). The direction of the unigenes was determined according to the best alignment results. The FPKM (reads per kb per million reads) value was used to estimate the gene expression level and was calculated as previously described^52^. Blast results of the *de novo* assemblies were used to retrieve Gene Ontology (GO) terms with Blast2GO^53^ under different categories: biological processes, molecular function and cellular component, which are hierarchically organized into different levels.

Alignments were performed using CLUSTALW v1.83 and the GeneDoc software^54^. A phylogenetic tree was constructed using the neighbor-joining method in MEGA6.0^55^ and visualized using TreeView^56^. The theoretical isoelectric points (pIs) and mass values of the mature peptides were calculated using the PeptideMass program (http://web.expasy.org/peptide_mass/).

### Construction of the SmNAC RNAi and SmNAC overexpression vectors and transformation procedures

We generated a 200 bp *SmNAC* forward fragment that was amplified by PCR (using the primers UP-P1: 5′-CGGATTTAAATGAAAATTAGAAAGATTACAAC-3′; DW-P1: 5′-CCGCCATGG TAAAAGAATATACATGTCCCT-3′), digested with *Swa*I and *Nco*I, and ligated into the pFGC5941 vector. The reverse fragment was amplified by PCR (using the primers UP-P2: 5′-CATGGATCCGAAAATTAGAAAGATTACAAC-3′; DW-P2: 5′-CTACCCGGGTAAAAGAA TATACA TGT CCCT-3′), digested with *Sma*I and *BamH*I and ligated into the pFGC5941 vector to create the RNAi-*SmNAC* vector. This binary vector was introduced into *Agrobacterium tumefaciens* strain EHA105. The PCR protocol used was as follows: 94 °C for 2 min, followed by 35 cycles of denaturation at 95 °C for 20 s, annealing at 55 °C for 30 s, and extension at 72 °C for 2 min. The RNAi-*SmNAC* vector then was transformed into ‘E-32’ eggplant and generated the RNAi-*SmNAC* transgenic plants.

The open reading frame (ORF) for S*mNAC* was amplified by PCR (5′-ATGGGTGTTCAAGAAAAAGAT CCTC-3′ and 5′-CTACTGTCTGAACCCGAGATTTAACGT-3′) and cloned into the pMD-T19 vector. The gene fragment and the expression vector pBI121 were then digested with *Sma*I and *Sac*I, and the *SmNAC* fragment was ligated into the digested vector using T_4_ ligase. The resulting overexpression vector, pBI-*SmNAC*, containing the CaMV35S promoter, Nos terminator, and the *NPT*-II gene, was then transformed into ‘E-31’ eggplant, using the methods as previously described^57^. Kanamycin (15 mg/L) was included in the growth medium to select for positive transformants, and resistant buds were obtained after 25 days. The presence of the transgene was verified by Southern blot analysis. T_1_ transgenic eggplant progeny were obtained following the self-pollination of T_0_ transgenic plants, and the transgenic T_1_ lines were tested for BW-resistance. Bacterial wilt growth was measured in transgenic eggplants as described by Zipfel *et al*.^58^. Briefly, using the syringe inoculation method, bacteria wilt was scraped off a fresh plate, resuspended in sterile water to 10^5^ colony-forming units (c.f.u.) ml^−1^, and pressure infiltrated into leaves with a needleless syringe. After 7 days, leaves were harvested and surface sterilized (30 s in 70% ethanol, followed by 30 s in sterile distilled water) for the spray inoculation method. Leaf discs from different leaves were ground in 10 mM MgCl_2_ using a microfuge tube glass pestle. After homogenization, the samples were thoroughly vortex-mixed and diluted 1:10 serially. Samples were finally plated on TZC solid medium (3 g casein hydrolysate, 5 g peptone, and 10 g glucose (pH 7.0). The plates were incubated at 28 °C for 2 days, after which the colony-forming units were counted.

### Immunohistochemical analysis of *SmNAC*

Fresh ‘E-32’ *S. melongena* stem and root tissues, and *SmNAC*-overexpressing and *SmNAC*-RNAi transgenic plants’ stem were cut into 4 mm pieces and fixed by vacuum infiltration for 2.5 h under ambient pressure in 3% paraformaldehyde, 0.1% Triton X-100 in phosphate buffered saline (PBS) at pH 7.2. The samples were dehydrated by incubation in increasing concentrations of 10%, 30%, 50%, 70% to 95% ethanol for 30 mins at each step, and placed in a polyethylene glycol (PEG) solution containing PEG1500:4000 (2:1) at 55 °C. After cooling at 4 °C, embedded material was sliced into 10 μm sections using a microtome. The sections were transferred to PBS to remove the PEG, washed for 10 min in PBS (pH 7.2), 5 min in 0.1 M NH_4_Cl, and then 5 min in PBS (pH 7.2). The tissues were next incubated in a blocking buffer consisting of 5% bovine serum albumin (BSA) in PBS (pH 7.2) for 1 h, then labeled with a polyclonal anti-*SmNAC* being generated by Invitrogen (Shanghai, China) or rabbit pre-immune serum for 12 h at 4 °C. The tissue sections were then washed three times with 0.1% BSA in PBS and immersed in a solution with anti-rabbit IgG conjugated to Alexa-Fluor 488 (Invitrogen, USA) at 37 °C for 2 h. After 4 washes with PBS, tissue sections were visualized using a reflected light microscope (Zeiss, Axioskop, Germany) with a dichroic filter (460–490 nm), and fluorescent images were acquired.

### Quantitative reverse-transcription (qRT-PCR) analysis

Quantitative reverse-transcription PCR (qPCR) was performed using gene-specific primers (Table S1) and a SYBR Premix Ex Taq kit (TaKaRa, Dalian, China), following the manufacturer’s protocols. The qRT-PCR cycles were as follows: an initial denaturation at 95 °C for 2 min, followed by 40 cycles of denaturation at 95 °C for 10 s, annealing at 56 °C for 20 s, and extension at 72 °C for 35 s. Triplicate qPCR reactions were performed for each sample, and the relative gene expression data were analyzed using the 2^−ΔΔ^Ct method^59^.

### Southern blot and Western blot analysis

DNA was extracted from leaves of the putative transgenic eggplant lines using the CTAB method, and Southern blot analysis was performed using a DNA labellling and detection kit (Boehringer Mannheim, Germany). The primers for the *NPT*-II probe used were F: 5′-TCGGCTATGACTGGGCACA-3′ and R: 5′-GATACCGTAAAGCACGAGGAAG-3′. The *Bar* primer sequences used were pBar1: 5′-ATGAGCCCAGAACGACGC-3′ and pBar2: 5′-TCT CAAATCTCGGTGACG-3. The PCR cycles were as follows: 5 min at 94 °C, followed by 35 cycles of 1 min at 94 °C, 1 min at 56 °C, 2 min at 72 °C, and one final 10 min step at 72 °C. The products were stored at 4 °C.

For Western-blot analysis, 30 μg of total proteins from plant tissue was separated by 10% SDS/PAGE gels and electro blotted onto a nitrocellulose membrane (GE Healthcare, Munich, Germany), and the membranes were preblocked with Tris-buffered saline (20 mM Tris, pH 7.5, and 150 mM NaCl) containing 5% (w/v) skim milk powder and 0.01% (v/v) Tween20. The membranes were probed with a primary anti-*SmNAC*, followed by a secondary goat anti-rabbit IgG antibody (Bio-Rad). Signals were detected using the ECL Prime Western Blotting Detection Reagent following the manufacturer’s protocols (GE Healthcare, Munich, Germany).

### Salicylic acid (SA) and jasmonic acid (JA) analysis

After inoculation, free SA was detected in *R. solanacearum* by HPLC at 0, 2, 4, 6 and 8 d, as previously described^60^. Quantitative determination of JA content was performed following the protocol described by Zhang *et al*.^61^. Each treatment repeated 3 times, and statistical analysis was used by the SPSS software.

### Subcellular localization of *SmNAC*

The coding region of *Sm-NAC*, without the stop codon, was amplified by PCR with the sub-*SmNAC*F (5′-ATGGGTGTTCAAGAAAAAGATCC-3′) and sub-*SmNAC* R (5′-CTACTGTCTGAACCCGAGATTTAAC-3′) primers. The PCR products were sub-cloned into the pEZS-NL-GFP vector in-frame with the green fluorescent protein (GFP) sequence, resulting in *SmNAC*-GFP vectors under the control of a CaMV 35S promoter. The fusion construct vector and the control GFP-alone vector were introduced into *S. melongena* (E-32) protoplasts using 40% PEG solution as previously described^62^. GFP fluorescence was visualized using a laser scanning confocal microscope (Leica TCS SP2, Leica Microsystems, Wetzlar, Germany). All transient expression assays were repeated at least three times.

### Promoter isolation

Genomic DNA was extracted from fresh ‘E-32’ (S) *S. melongena* leaves using the DNeasy Plant Mini Kit (Qiagen, Germany). The promoter of the eggplant isochorismate synthase 1 (*ICS1*) gene was isolated using a Genome Walker Kit (Clontech, USA) by nested PCR (primers shown in Table S2) according to the manufacturer’s protocols. After sequencing, conserved *cis*-element motifs of the promoter were predicted using the PLACE (http://www.dna.affrc.go.jp/PLACE/signalscan.html) and Plant-CARE (http://bioinformatics.psb.ugent.be/webtools/plantcare/html/) databases.

### Protoplast transformation assay

For transactivation analysis of *SmNAC*, the *SmNAC* coding sequence without the stop codon was cloned into the reconstructed GAL4-DBD vector (Clontech, USA), producing the effector construct. The double reporter vectors were kindly provided by Professor Jianye Chen (Horticultural College of South Agricultural University) and included a GAL4-LUC and an internal control, REN, driven by the 35S promoter. GAL4-LUC contains five copies of a GAL4-binding element and a minimal TATA region of the 35S promoter, which are all located upstream of the *LUC* gene. To assay the binding activity of *SmNAC* to the *ICS*1 promoter, the *ICS*1 promoter was cloned into a pGreenII 0800-LUC double reporter vector, whereas *SmNAC* was inserted into the pGreenII 62-SK vector, generating the effector construct^63^. The effector and reporter plasmids were co-transformed into *S. melongena* protoplasts as previously described^64^ and incubated as described above. LUC and REN luciferase activities were measured using a dual luciferase assay kit (Promega, USA). The analysis was performed using the Luminoskan Ascent Microplate Luminometer (Thermo, USA) with a 5 s delay and 15 s integration time. The binding activity of *SmNAC* to the *ICS1* promoter was measured as a ratio of LUC to REN. At least six transient assays were measured for each assay.

### Yeast One-Hybrid assay

The sequence encoding the *SmNAC* domain (amino acids 1-345) was inserted into pGADT7 (pAD) vector (Clontech, USA) to generate the pAD-*SmNAC* construct. Six DNA fragments of the eggplant *ICS*1 promoter (−1 to −370 bp, −380 to −520 bp, −550 to −750 bp, −952 to −1170 bp, −1180 to −1318 bp, and −1410 to −1570 bp) were ligated into the pAbAi vector (Clontech, USA). Transformation of the Y1H yeast strain AH109 with these pAbAi vectors containing the promoter fragments yielded the bait yeast strains, which were further transfected with pAD-*SmNAC*. Growth of the transformants on SD/-Leu/+AbA medium was considered as an indicator for *SmNAC* binding to the corresponding DNA fragment.

### Statistical Analysis

Data were expressed as mean ± standard deviation (SD). Statistical analysis was used by the SPSS (Statistical Product and Service Solutions) software. A value of p < 0.05, p < 0.01 was considered to be statistically significant. Statistical analysis was performed with Excel 2010.

## Additional Information

**How to cite this article**: Na, C. *et al*. Overexpression of the Eggplant (*Solanum melongena*) NAC Family Transcription Factor *SmNAC* Suppresses Resistance to Bacterial Wilt. *Sci. Rep.* **6**, 31568; doi: 10.1038/srep31568 (2016).

## Supplementary Material

Supplementary Information

## Figures and Tables

**Table 1:** Evaluation of bacterial wilt resistance in *SmNAC* overexpressing plants.

Material	Generation	Evaluation of scale				
		0	1	2	3	4
EGT_0–5_	T_0_	0	1	2	22	5
EGT_0–23_	T_0_	0	2	3	17	8
EGT_0–43_	T_0_	0	2	5	21	2
EGT_0–87_	T_0_	0	0	4	20	6
EGT_0–145_	T_0_	0	0	5	20	5
EGT_0–204_	T_0_	0	1	3	23	3
EGT_1–87_	T_1_	0	0	3	25	3
EGT_1–145_	T_1_	0	0	1	23	6
EGT_1–204_	T_1_	0	0	4	18	8
E-31	CK	14	12	4	0	0

Evaluation of scale: 0 = healthy, 1 = one or two leaves wilted, 2 = three or more leaves wilted, 3 = all leaves wilted, and 4 = dead. Grades 0–2 were considered bacterial wilt resistant, and grades 3–4 susceptible. Bacterial wilt resistance of all plants was assessed after 20 dpi.

**Table 2:** Evaluation of bacterial wilt resistance of RNAi-*SmNAC* transgenic plants.

Material	Generation	Evaluation of scale				
		0	1	2	3	4
RNAi-1	T_0_	1	4	19	6	0
RNAi-2	T_0_	1	3	22	5	0
RNAi-3	T_0_	2	3	20	5	0
RNAi-4	T_0_	1	4	22	3	0
RNAi-5	T_0_	2	5	18	5	0
RNAi-1-1	T_1_	2	12	15	10	0
RNAi-2-1	T_1_	3	15	17	12	0
RNAi-3-1	T_1_	3	14	18	10	1
E-32	CK	0	0	3	15	18

Evaluation scale: 0 = healthy, 1 = one or two leaves wilted, 2 = three or more leaves wilted, 3 = all leaves wilted, and 4 = dead. Grades 0–2 were considered bacterial wilt resistant, and grades 3–4 susceptible. Bacterial wilt resistance of all plants was assessed after 20 dpi.

**Table 3:** Salicylic acid content of transgenic eggplants after inoculation (μg.g^−1^ fresh weight).

Lines	Generation	Time of treatment (d)				
		0	2	4	6	8
EGT_0–5_	T_0_	2.83 ± 0.32b	2.15 ± 0.24B	1.57 ± 0.11B	0.74 ± 0.02B	0.28 ± 0.01B
EGT_0–23_	T_0_	2.72 ± 0.17b	2.07 ± 0.12B	1.61 ± 0.08B	0.81 ± 0.02B	0.23 ± 0.01B
EGT_0–43_	T_0_	2.64 ± 0.25b	2.13 ± 0.11B	1.57 ± 0.09B	0.94 ± 0.03B	0.31 ± 0.02B
EGT_0–87_	T_0_	2.55 ± 0.23b	1.95 ± 0.19B	1.41 ± 0.03B	0.73 ± 0.05B	0.25 ± 0.01B
EGT_0–145_	T_0_	2.87 ± 0.22b	2.34 ± 0.15B	1.54 ± 0.02B	0.85 ± 0.03B	0.36 ± 0.03B
EGT_0–204_	T_0_	2.91 ± 0.19b	2.04 ± 0.12B	1.46 ± 0.05B	0.76 ± 0.01B	0.18 ± 0.01B
EGT_1–87_	T_1_	2.87 ± 0.21b	2.15 ± 0.14B	1.71 ± 0.01B	0.81 ± 0.06B	0.22 ± 0.02B
EGT_1–145_	T_1_	2.81 ± 0.13b	2.06 ± 0.11B	1.63 ± 0.11B	0.75 ± 0.03B	0.27 ± 0.01B
EGT_1–204_	T_1_	2.68 ± 0.24b	1.93 ± 0.18B	1.33 ± 0.11B	0.67 ± 0.01B	0.21 ± 0.01B
E-31	CK_1_	4.76 ± 0.25a	6.68 ± 0.44 A	8.82 ± 0.39A	13.67 ± 0.87A	9.58 ± 0.75A
RNAi-1	T_0_	4.15 ± 0.34a	5.62 ± 0.39A	7.64 ± 0.63a	10.35 ± 0.84A	8.42 ± 0.61A
RNAi-2	T_0_	4.23 ± 0.26a	5.04 ± 0.19A	7.21 ± 0.58a	11.21 ± 0.89A	8.04 ± 0.52A
RNAi-3	T_0_	4.56 ± 0.41a	6.13 ± 0.24A	7.54 ± 0.47a	10.64 ± 0.93A	7.95 ± 0.66A
RNAi-4	T_0_	4.31 ± 0.35a	6.15 ± 0.44A	7.27 ± 0.62a	10.83 ± 0.59A	7.33 ± 0.59A
RNAi-5	T_0_	4.47 ± 0.32a	5.97 ± 0.43A	7.18 ± 0.49a	11.33 ± 0.67A	7.61 ± 0.45A
RNAi-1-1	T_1_	4.53 ± 0.32a	6.21 ± 0.29A	7.37 ± 0.53a	10.87 ± 0.88A	6.95 ± 0.55A
RNAi-2-1	T_1_	4.67 ± 0.27a	6.25 ± 0.35A	7.55 ± 0.53a	11.24 ± 0.25A	7.41 ± 0.51A
RNAi-3-1	T_1_	4.36 ± 0.24a	6.22 ± 0.33A	7.46 ± 0.54a	11.62 ± 0.37A	7.46 ± 0.61A
E-32	CK_2_	3.87 ± 0.29b	4.52 ± 0.31B	6.12 ± 0.54b	4.33 ± 0.23B	2.65 ± 0.25B

Error bars represent standard error. ab, AB, Significantly different from CK: ab, P < 0.05; AB, P < 0.01.

**Table 4:** Jasmonic acid content in transgenic eggplant after inoculation pathology (μg.g^−1^ fresh weight).

Lines	Generation	Time of treatment (d)				
		0	2	4	6	8
EGT_0–5_	T_0_	4.23 ± 0.12	4.85 ± 0.22a	5.57 ± 0.32A	7.14 ± 0.24A	6.78 ± 0.21A
EGT_0–23_	T_0_	4.22 ± 0.14	4.87 ± 0.13a	5.61 ± 0.31A	7.21 ± 0.22A	6.73 ± 0.33A
EGT_0–43_	T_0_	4.34 ± 0.22	4.83 ± 0.11a	5.67 ± 0.24A	7.24 ± 0.25A	6.81 ± 0.24A
EGT_0–87_	T_0_	4.15 ± 0.23	4.75 ± 0.22a	5.64 ± 0.23A	7.23 ± 0.33A	6.75 ± 0.28A
EGT_0–145_	T_0_	4.27 ± 0.22	4.94 ± 0.15a	5.71 ± 0.32A	7.25 ± 0.23A	6.86 ± 0.24A
EGT_0–204_	T_0_	4.11 ± 0.19	4.91 ± 0.15a	5.66 ± 0.25A	7.16 ± 0.19A	7.06 ± 0.23A
EGT_1–87_	T_1_	4.12 ± 0.17	4.85 ± 0.17a	5.81 ± 0.21A	7.21 ± 0.26A	6.92 ± 0.12A
EGT_1–145_	T_1_	4.21 ± 0.15	4.86 ± 0.28a	5.93 ± 0.28A	7.15 ± 0.23A	6.82 ± 0.27A
EGT_1–204_	T_1_	4.28 ± 0.23	4.93 ± 0.28a	5.83 ± 0.21A	7.17 ± 0.24A	6.88 ± 0.29A
E-31	CK_1_	4.16 ± 0.26	3.18 ± 0.31b	2.72 ± 0.18B	1.38 ± 0.27B	0.75 ± 0.05B
RNAi-1	T_0_	5.95 ± 0.14	3.92 ± 0.19	2.64 ± 0.13B	1.95 ± 0.14B	1.32 ± 0.01B
RNAi-2	T_0_	5.98 ± 0.16	4.04 ± 0.21	2.51 ± 0.24B	1.97 ± 0.28B	1.38 ± 0.11B
RNAi-3	T_0_	5.96 ± 0.24	3.93 ± 0.14	2.54 ± 0.27B	1.94 ± 0.33B	1.35 ± 0.11B
RNAi-4	T_0_	5.91 ± 0.25	3.95 ± 0.23	2.67 ± 0.28B	1.98 ± 0.18B	1.33 ± 0.10B
RNAi-5	T_0_	5.93 ± 0.22	3.97 ± 0.31	2.62 ± 0.19B	1.95 ± 0.25B	1.36 ± 0.07B
RNAi-1-1	T_1_	5.97 ± 0.12	3.96 ± 0.22	2.57 ± 0.23B	1.97 ± 0.28B	1.35 ± 0.08B
RNAi-2-1	T_1_	6.07 ± 0.17	4.05 ± 0.13	2.55 ± 0.15B	2.02 ± 0.25B	1.37 ± 0.07B
RNAi-3-1	T_1_	6.02 ± 0.21	3.95 ± 0.33	2.58 ± 0.14B	1.96 ± 0.36B	1.31 ± 0.02B
E-32	CK_2_	5.97 ± 0.17	4.02 ± 0.31	5.95 ± 0.31A	7.97 ± 0.27A	6.41 ± 0.28A

Error bars represent standard error. ab, AB, Significantly different from CK: ab, P < 0.05; AB, P < 0.01.

**Table 5:** Effect of exogenous salicylic acid on bacterial wilt resistance in *SmNAC* overexpressing plants.

Material	#of plants	Treatment	% of plants with wilt symptoms after inoculation			
			7 dpi	10 dpi	15 dpi	20 dpi
EGT_1–204_	60	CK	38.3	81.7	100	
	60	SA treatment	0	18.3	61.7	100
E-32	60	CK	55.0	86.7	100	
	60	SA treatment	0	26.7	66.7	100

CK: plants were sprayed with water, before inoculation with the pathogen. SA (salicylic acid) treatment: plants were treated with 0.2 mM SA before inoculation with the pathogen. dpi, days post-inoculation.
