# Supplementary material for: Overexpression of the Eggplant (Solanum melongena) NAC Family Transcription Factor SmNAC Suppresses Resistance to Bacterial Wilt
Source: Sci Rep. 2016 Aug 16;6:31568. doi: 10.1038/srep31568 (PMC4985710; doi:10.1038/srep31568)
Supplement: Supplementary Information [file srep31568-s1.pdf]

The title page

The manuscript title:

Overexpression of the Eggplant (*Solanum melongena*) NAC Family Transcription Factor, S*mNAC*, Suppresses Resistance to Bacterial Wilt.

The author list:

1 Cao Bihao: *Corresponding author
Institution : College of Horticulture, South China Agricultural University
Postcode: 510642
Telephone: +86-2085280228
Fax: +86-20-85282107
E-mail: caobh01@163.com.

2 Chen Na: The first author
Institution: College of Horticulture, South China Agricultural University
Postcode: 510642
E-mail: 976210387@qq.com

3 Wu Shuanghua: The second author
Institution: College of Horticulture, South China Agricultural University
Postcode: 510642
E-mail: 398573280@qq.com

4 Fu Jinglong: The third author
Institution: College of Horticulture, South China Agricultural University
Postcode: 510642
E-mail: 1007674018@qq.com

5 Lei Jianjun: The fifth author
Institution : College of Horticulture, South China Agricultural University
Postcode: 510642, P.R.China
E-mail: jjlei@scau.edu.cn

6 Chen Changming: The sixth author
Institution: College of Horticulture, South China Agricultural University
Postcode: 510642
E-mail: 104845357@qq.com

7 Jiang Jin: The seventh author
Institution: College of Horticulture, South China Agricultural University
Postcode: 510642
E-mail: 921571335@qq.com

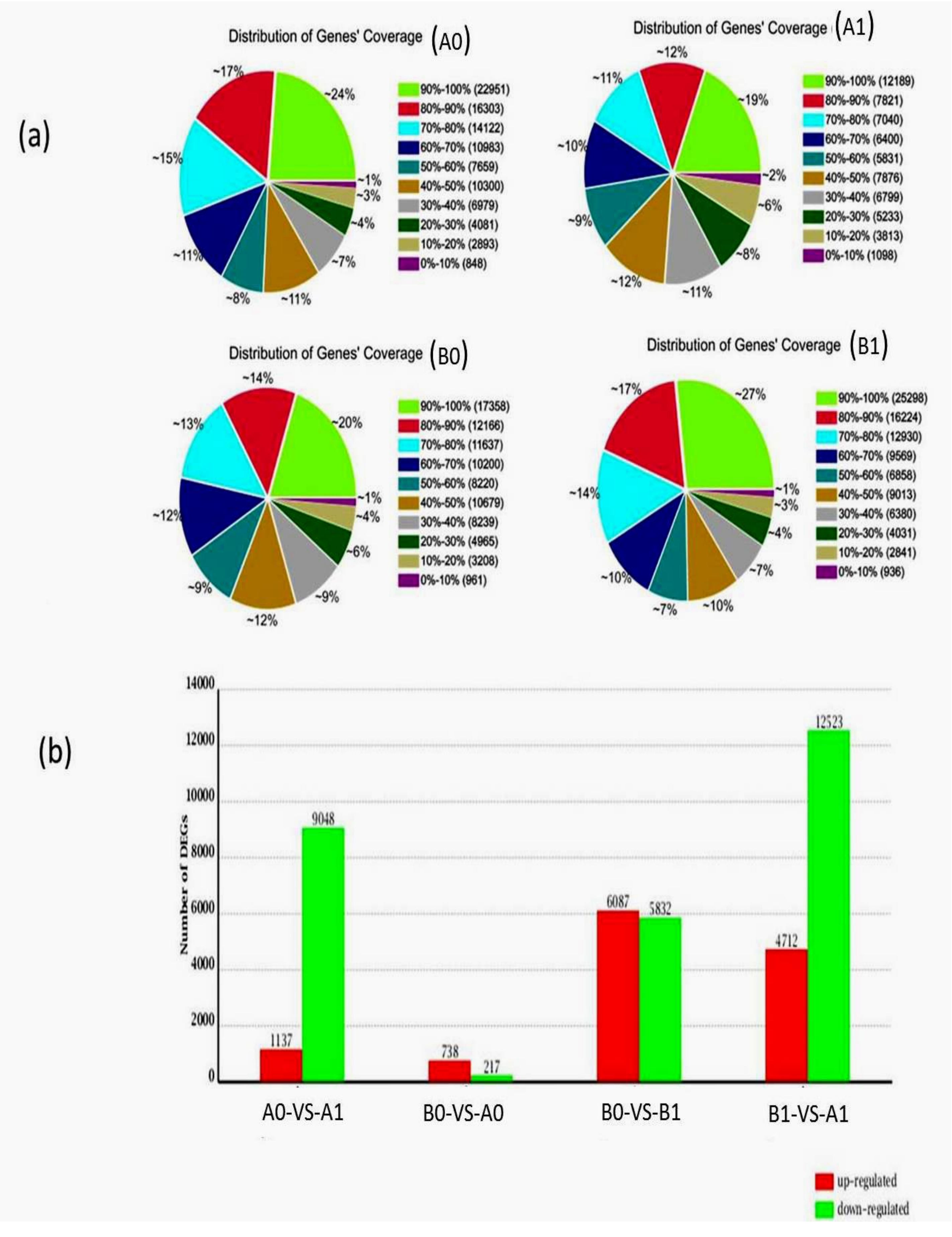

(a)
Distribution of Genes' Coverage (A0)
~24%
~17%
~15%
~11%
~8%
~11%
~7%
~4%
~3%
~1%
90%-100% (22951)
80%-90% (16303)
70%-80% (14122)
60%-70% (10983)
50%-60% (7659)
40%-50% (10300)
30%-40% (6979)
20%-30% (4081)
10%-20% (2893)
0%-10% (848)
Distribution of Genes' Coverage (A1)
~12%
~11%
~19%
~10%
~2%
~6%
~9%
~8%
~12%
~11%
90%-100% (12189)
80%-90% (7821)
70%-80% (7040)
60%-70% (6400)
50%-60% (5831)
40%-50% (7876)
30%-40% (6799)
20%-30% (5233)
10%-20% (3813)
0%-10% (1098)
Distribution of Genes' Coverage (B0)
~14%
~13%
~20%
~12%
~1%
~4%
~6%
~9%
~9%
~12%
90%-100% (17358)
80%-90% (12166)
70%-80% (11637)
60%-70% (10200)
50%-60% (8220)
40%-50% (10679)
30%-40% (8239)
20%-30% (4965)
10%-20% (3208)
0%-10% (961)
Distribution of Genes' Coverage (B1)
~17%
~27%
~14%
~1%
~3%
~4%
~7%
~10%
~7%
~10%
90%-100% (25298)
80%-90% (16224)
70%-80% (12930)
60%-70% (9569)
50%-60% (6858)
40%-50% (9013)
30%-40% (6380)
20%-30% (4031)
10%-20% (2841)
0%-10% (936)
(b)
Number of DEGs
14000
12000
10000
8000
6000
4000
2000
0
1137
9048
738
217
6087
5832
4712
12523
A0-VS-A1
B0-VS-A0
B0-VS-B1
B1-VS-A1
up-regulated
down-regulated

(c)

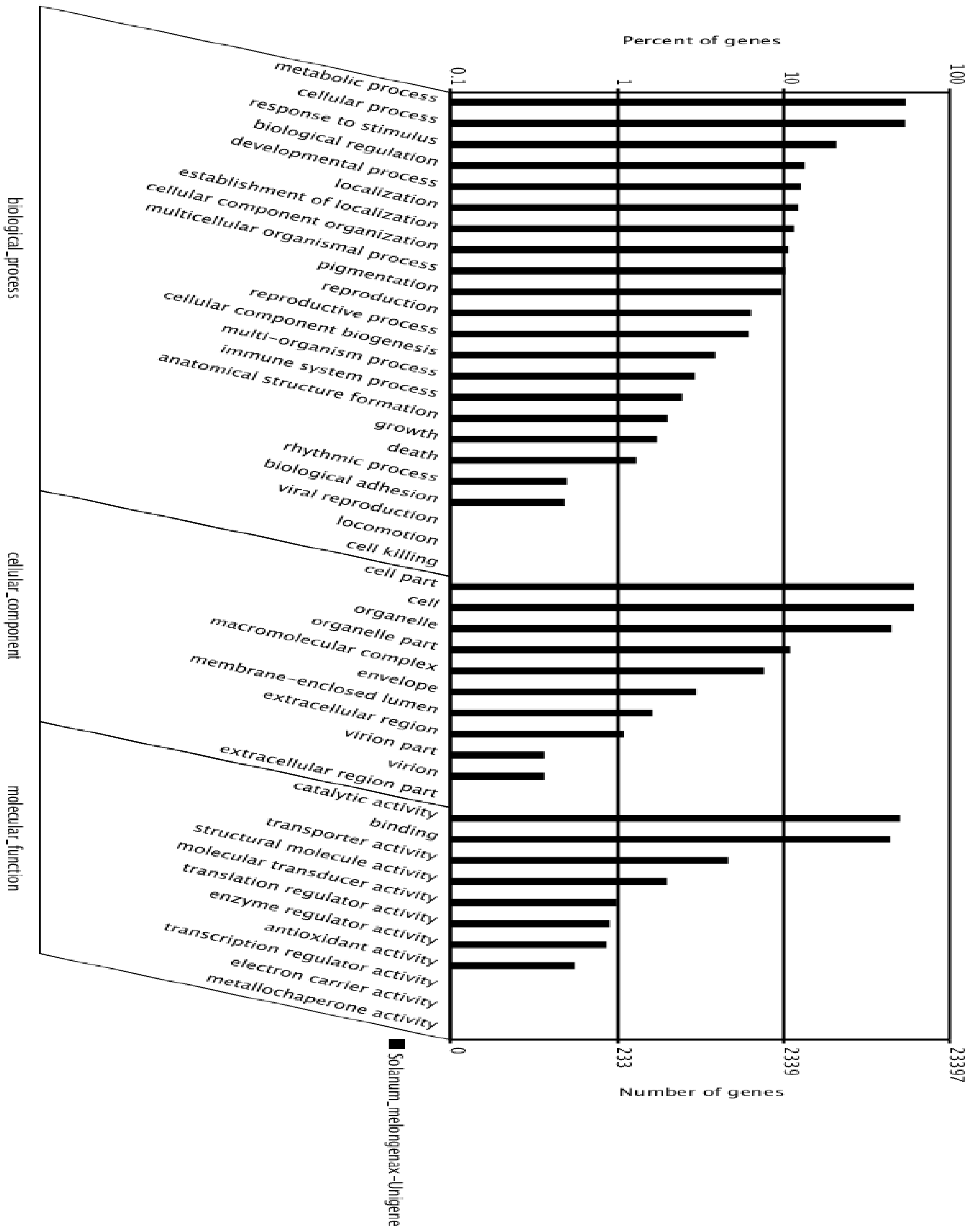

Fig.S1. Distribution of gene coverage in each RNA-seq library, Differential gene expression analysis and GO-terms.

RNA-Seq analysis was performed on resistant (E-31) and susceptible (E-32) eggplant lines. Lines A0 and A1 corresponded to mock-inoculated and pathogen-inoculated E-31 plants at 7 days post-inoculation (dpi). Similarly, B0 and B1 correspond to mock-inoculated and pathogen-inoculated E-32 plants at also 7 dpi. RNA was extracted from ten plants stems of each of these four lines and used for RNA-Seq library construction, with 2 replicates for each treatment.

(a) Distribution of gene coverage of each library. (b) Differential gene expression analysis.

(c) Frequency of sequences with assigned Gene Ontology (GO) terms (slimmed) for the category biological process found in the stem tissue assemblies.

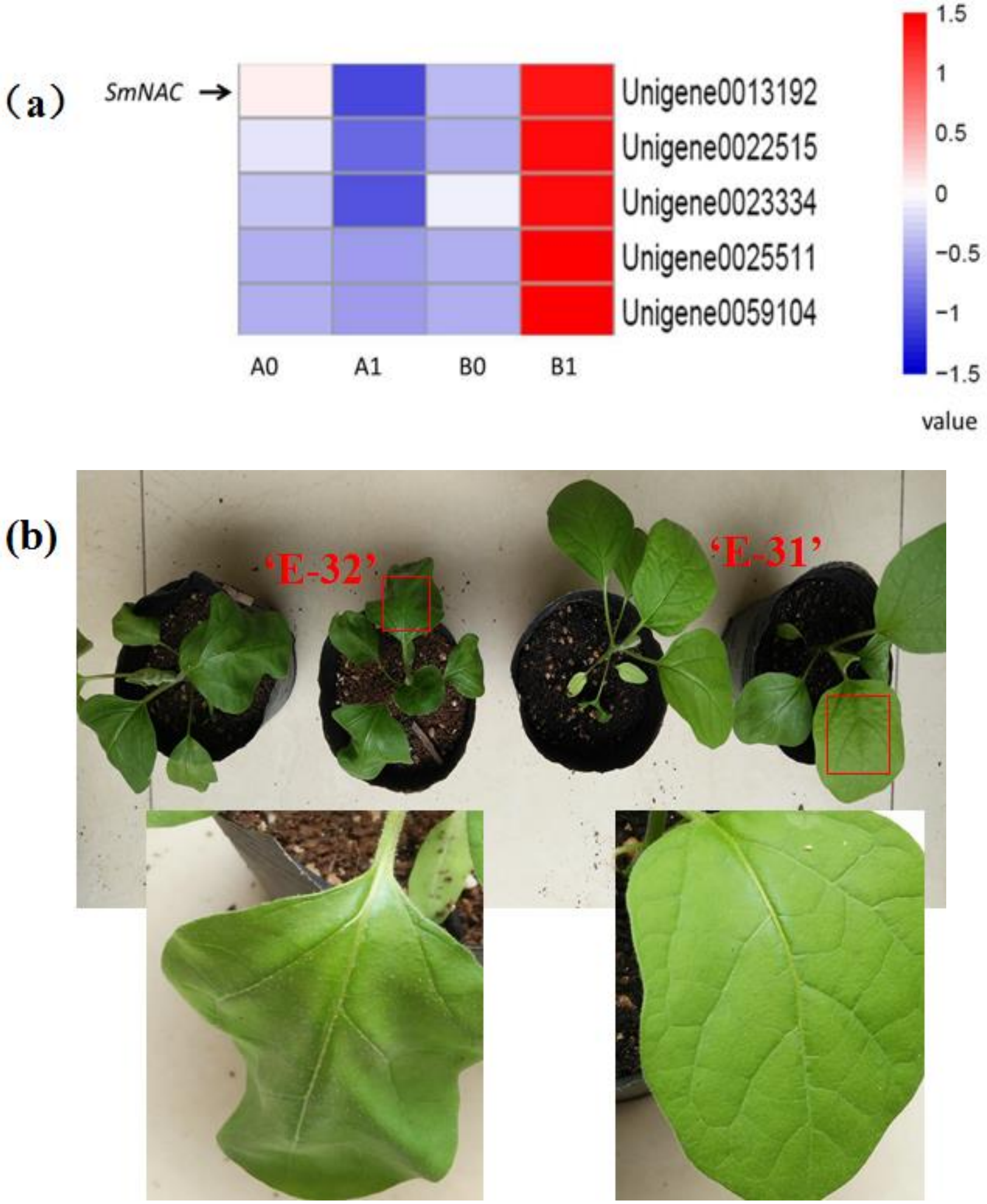

Fig.S2. The heat maps depict the expression patterns of *SmNAC* in resistant plant and susceptible plant, and the phenotypic symptoms of susceptible plants after inoculation with *Ralstonia solanacearum*.

(a): The heat maps depict the expression patterns of *SmNAC* in resistant plant and susceptible plant
Heat-map values represent log-transformed relative expression values.

A0 and A1 corresponded to mock-inoculated and pathogen-inoculated E-31 plants, 7 days post-inoculation (dpi). B0 and B1 correspond to mock-inoculated and pathogen-inoculated E-32 plants, also 7 dpi. Arrow showing the expression level of *SmNAC*.

(b): The phenotypic symptoms of eggplants after 15 days inoculation with *Ralstonia solanacearum*.
'E-32': susceptible plant; 'E-31': resistant plant.

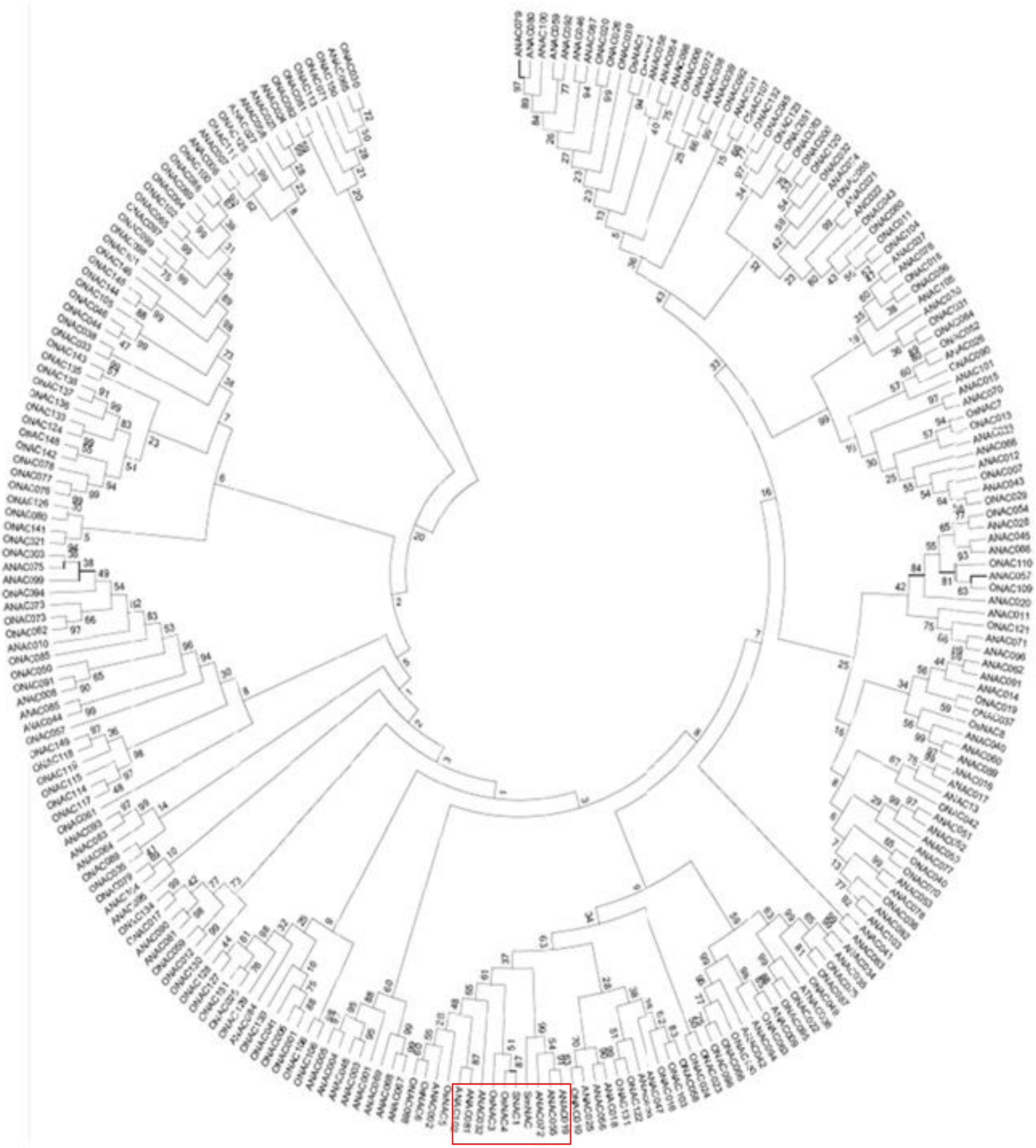

Fig.S3. A phylogenetic tree including *SmNAC* and related proteins.

Phylogenetic tree of NAC proteins (black circles). Protein sequences were aligned with those of the *A. thaliana* and rice NAC families. A multiple sequence alignment was carried out using CLUSTALW, and the phylogenetic tree was constructed in MEGA6.0 using the minimum evolution test and default parameters. Bootstrap values indicate the divergence of each branch and the scale indicates branch length. The full-length amino acid sequences of the ANAC proteins were obtained from the Plant Transcription Factor Database (http://planttfdb.cbi.pku.edu.cn/ family.php?sp=Ath&fam=NAC) and the full length amino acid sequences of the ONAC proteins were obtained from the Rice genome annotation project (http://rice.plantbiology.msu.edu). Full length sequences were used in the analysis.

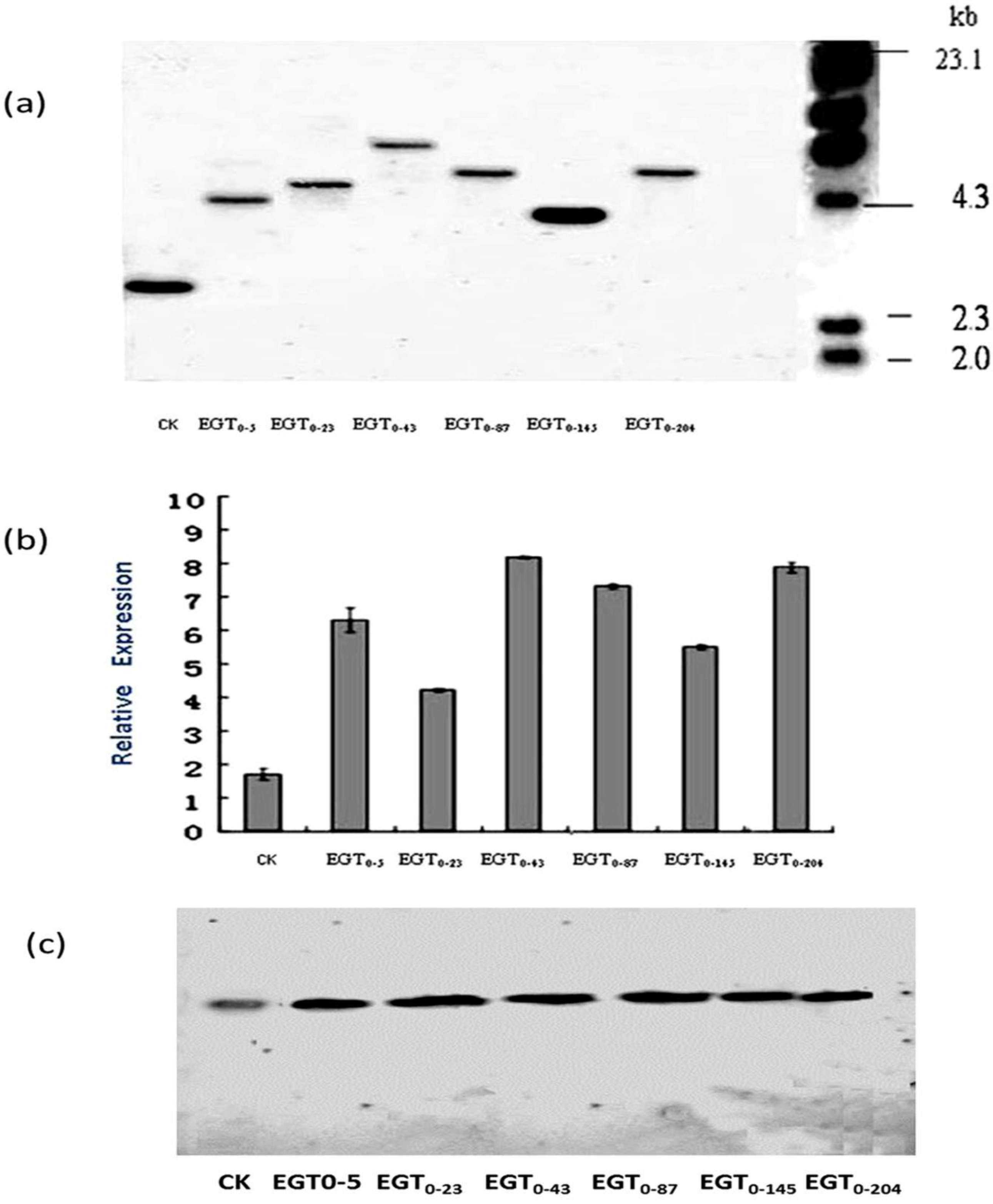

Fig.S4. Southern blot, qRT-PCR and Western blot analyses of *SmNAC* in *SmNAC*-overexpressing plants.

(a) Genomic DNA from transgenic Km-resistant plants was digested with *Hin*dIII, and a *NPT*-II gene fragment was used as a probe. CK: pBI-*SmNAC*. (b) qRT-PCR analysis of *SmNAC* expression in transgenic plants using the primers: F-P1 5’-GAAAATTAGAAAGATTACAAC-3’ and R-P1 5’-TAAAAGAATATACATGTCCCT-3’. CK: Non-transgenic plant from the ‘E-31’ line. (c) Western blot detection of *SmNAC* in overexpression transgenic plants, anti-*SmNAC* was used as the probe. CK: Non-transgenic plant from the ‘E-31’ line; EGT$_{0-5}$, EGT$_{0-23}$, EGT$_{0-43}$, EGT$_{0-87}$, EGT$_{0-145}$, and EGT$_{0-204}$ represent the *SmNAC*-overexpression plants.

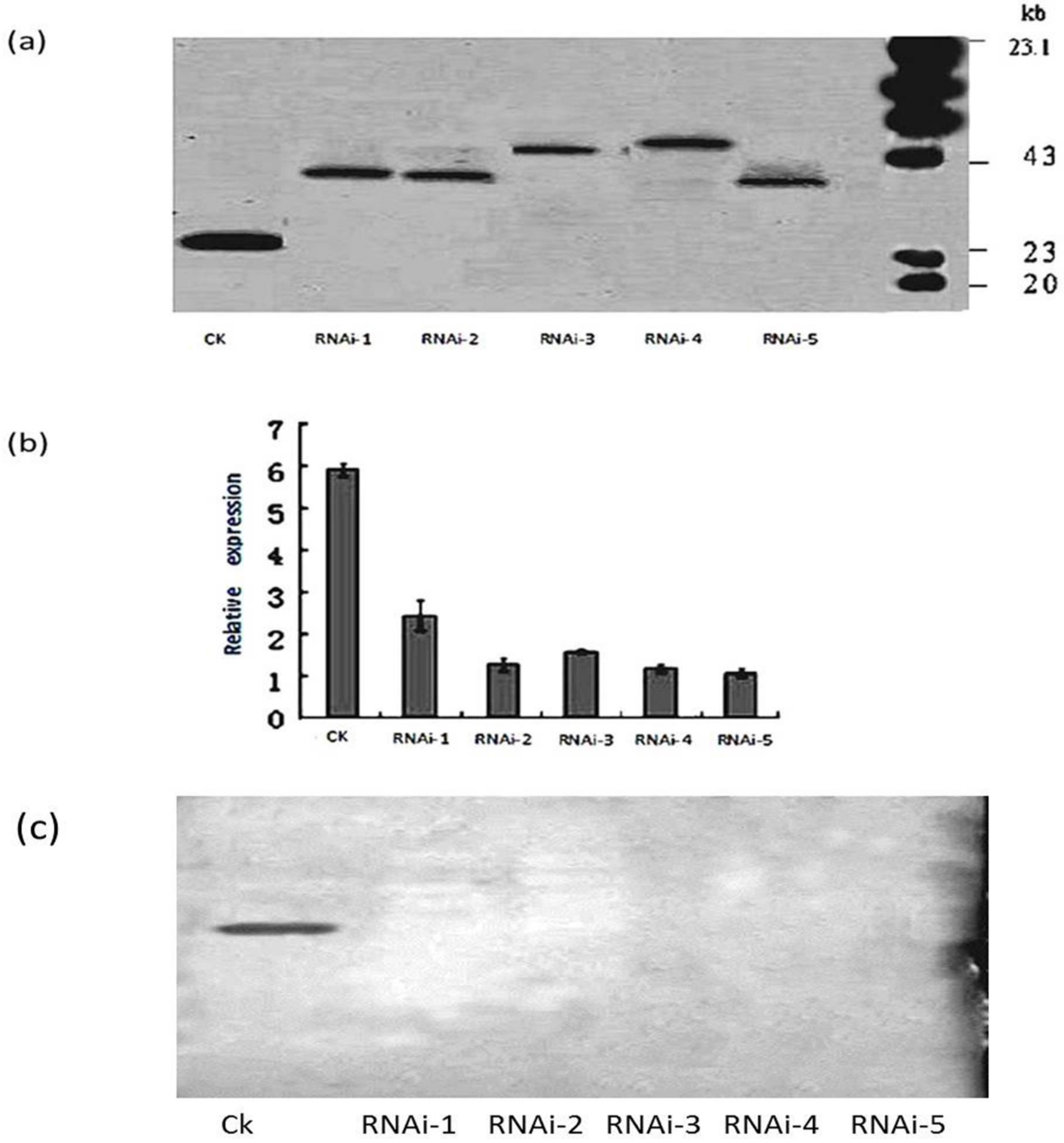

Fig.S5. Southern blot, qRT-PCR and Western blot analysis of expression of *SmNAC* in RNAi-*SmNAC* plants.

(a) Genomic DNA from the resistant transgenic plants was digested with *Hin*d III, and *Bar* gene fragments were used as a probe. CK: pFGC5941-*SmNAC*. (b) qRT -PCR analysis of *SmNAC* in transgenic plants using the primers: F-P1 5'-GAAAATTAGAAAGATTACAAC-3' and R-P1 5'-TAAAAGAATATACA TGTCCCT-3'. CK: Non-transgenic plants from the 'E-32' line. (c) Western blot detection of *SmNAC* RNAi transgenic plant, anti-*SmNAC* was used as the probe. CK: Non-transgenic plants from the 'E-32' line; RNAi-1, RNAi-2, RNAi-3, RNAi-4, and RNAi-5 represent the *SmNAC*-RNAi transgenic plants.

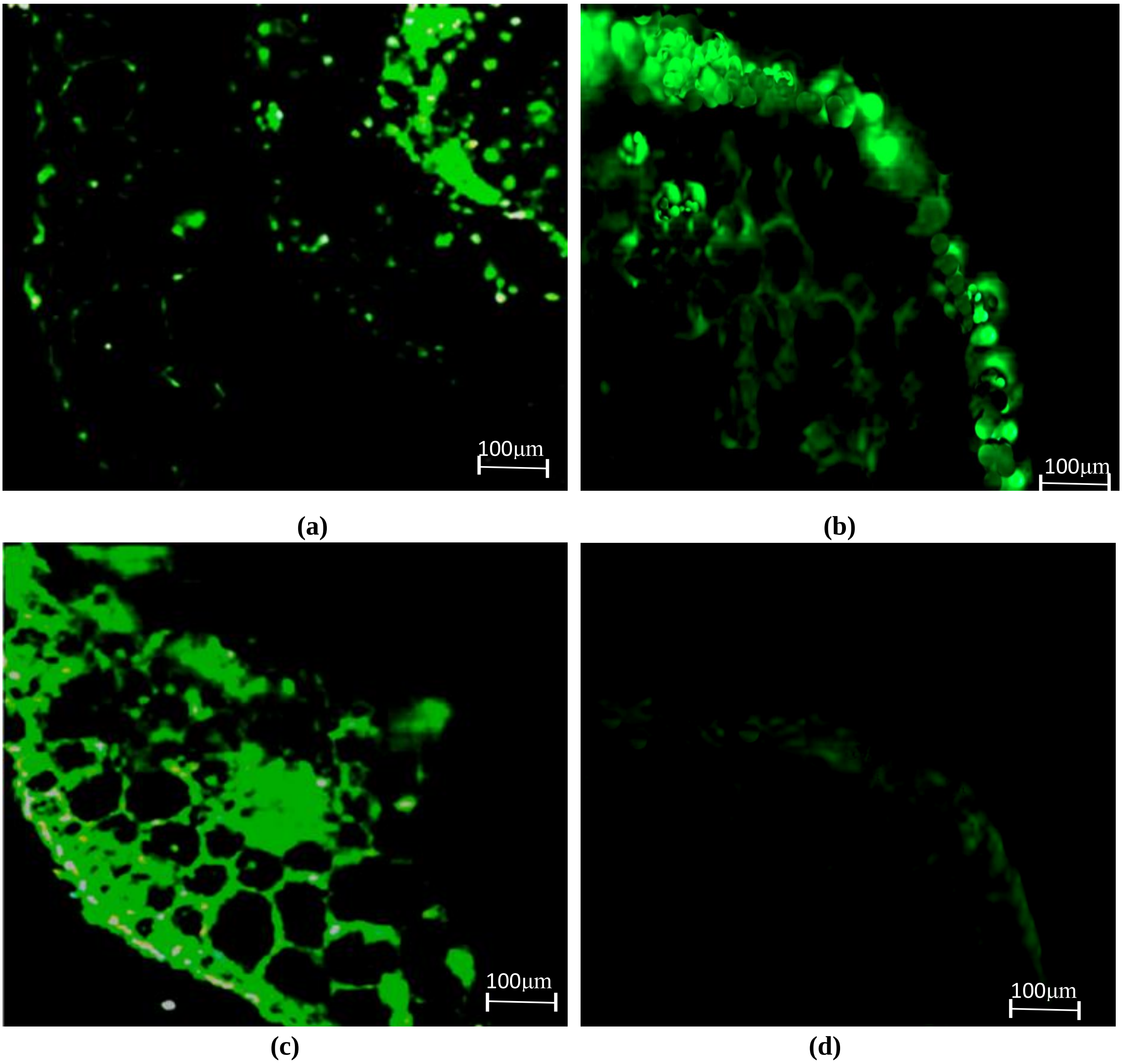

Fig.S6. Immunohistochemical localization analysis of *SmNAC* in *SmNAC*-overexpressing and *SmNAC*-RNAi transgenic plants.

Fresh *S. melongena* stem tissues were used, and cross-sections of eggplant tissue labeled with purified *SmNAC* antibodies and visualized using an anti-rabbit IgG conjugated to green fluorescent protein. Scale bars, 100μm. Each experiment repeated three times.

(a) *SmNAC* protein distribution in the stem of a non-transgenic plant (E-31);

(b) *SmNAC* protein distribution in the stem of a *SmNAC*-overexpressing plant ($EGT_{1\text{-}87}$);

(c) *SmNAC* protein distribution in the stem of a non-transgenic plant (E-32);

(d) *SmNAC* protein distribution in the stem of a *SmNAC*-RNAi transgenic plant (RNAi-2-1).

TableS1 primer sequence of qRT-PCR

Gene name	Forward(5'-3')	Reverse(5'-3')	Gen Bank accession
Pin2	TGATGCCAAGGCTTGTACTAGAGA	AGCGGACTTCCTTCTGAACGT	AY129402
JAR1	GCAGCTATGGGGTTTGAATGGCAGA	AAGAGGTCAGGAATCAAGCCGTACC	DQ359730.1
LoxA	GACCAAAACGCTCGTCTCTC	TGGTAGACCACCAACACGAA	U09026
EDS1	GTTTCGCAGACAAGTTGAGCC	CTCTGTGTGAACCGATAACGC	AY679160.1
GluA	GCCGACTGGGTGAGATGGTAA	ACATTGTTGTGCCCGTGGAC	M80604
NPR1	CTTGGACTGGGTGTTGCTAATG	TGCCCATCCAATGTAATGTCTG	NM_001247633.1
TGA	GCAAGTGACCCTGAACTACGAAG	GGGTTTTCCACATCCCTGACAAG	GQ386946.1
SGT1	TTCTCGGTTTTGAGGAAGGG	GCAGATACCAAGTGATGTCTACCA	NM_001247758.1
PAD4	ACATCGGCTGAAACCTCCTTATT	TTTGATAAGTGGTGGGGAAATGA	AY753546.1
PR-1a	GAGGGCAGCCGTGCAA	CACATTTTTCCACCAACACATTG	M69247
EIN2	GGGACGAACGGGCACTGCAGCA	GTGATGAGGCAGACAGCGTTACT	AY566238
EIL1	TTGAACGACAGCCCTGAGTATG	AGGTGTCCAGTTCTTCTTTGTTCG	NM_001247612.1
ICS1	GCATGGGACAATGCTGCTGCCTCATGGA	TCTGGTGCTACGAGCAAGTACCACCT	NM_001247865.1
PBS3	GTTTGCACTATGTATGCTTCCTCTG	GCTCATACTCCTGACCAATCTTtgtcGACA	XM_004251437.2
BSMT1	TATGAGTGGGAACTTTTTGGCTACGACGC	GCCATATCGTATTTCATCATCACAAG	XM_004237341.1
SAGT1	GCCTTCGTTTATTTACGTACATGGAACG	CCACATGGAATAGATTGAGACCATAC	XM_004245012.1
ACTIN	TATTGTGGGTCGTCCTCG	TCTCTCTGTTGGCCTTGG	XM_016658880.1

TableS2 Primer sequences used for promoter isolation

Primer	Sequence of primer
LAD1	5’-ACGATGGACTCCAGAGNTCGA(G/C)T(A/T)T(G/C)G(A/T)GTT-3’
LAD2	5’--ACGATGGACTCCAGAG NGTCGA(G/C)(A/T)GANA(A/T)GAA-3’
LAD3	5’--ACGATGGACTCCAGAG (A/T)TCTGNCT(A/T)ANTANCT-3’
LAD4	5’--ACGATGGACTCCAGAG AG(A/T)GNAG(A/T)ANCA(A/T)AGG-3’
AC1	5’-ACGATGGACTCCAGAG-3’
SP1	5’-AGGATCGGACTTAATCATATCGGAGATGGC-3’
SP2	5’-GCAGGGCTAGAAACTGCAGGCAAAGT-3’
SP3	5’-CCAATAGGAGCTCTTGAATCGCCTTGGCA-3’

TableS3 Main regulatory motifs found within the promoter sequence of *ICS*1 in eggplant

Factor or site name	Site	Signal sequence	Function
**ABRE**	167(+),1350(+)	CACGTG	cis-acting element involved in the abscisic acid responsiveness
**AE-box**	881(-)	AGAAACAT	part of a module for light response
**ARE**	1134(-)	TGGTTT	cis-acting regulatory element essential for the anaerobic induction
**ATCT-motif**	501(+)	AATCTAATCT	part of a conserved DNA module involved in light responsiveness
**Box-4**	343(+),265(+)	ATTAAT	part of a conserved DNA module involved in light responsiveness
**Box –I**	9(-),846(-), 615(+),1075(+)	TTTCAAA	light responsive element
**ERE**	125（-）,1389(-)	ATTTCAAA	ethylene-responsive element
**CAAT-box**	109(+),316(+) 471(+),936(+), 1138(+),1323(+)	CAAT	common cis-acting element in promoter and enhancer regions
**CAT-box**	1336(+)	GCCACT	cis-acting regulatory element related to meristem expression
**EIRE**	125(-),1389(-)	TTCGACC	elicitor-responsive element
**G-Box**	167(+), 956(+), 1350(+)	CACGTG, CACGTT	cis-acting regulatory element involved in light responsiveness
**HD-Zip 1**	37(+)	CAAT(A/T)ATTG	element involved in differentiation of the palisade mesophyll cells
**HSE**	73(+),998(+), , 1150(+)	AAAAAATTTC	cis-acting element involved in heat stressres ponsiveness
**Skn-1motif**	136(+),495(-)	GTCAT	cis-acting regulatory element required for endosperm expression
**TATA-box**	69(+),145(+), 242(+),386(+),489(+), 642(+),789(-),908(-), 116(-),1163(+)	TATAA	core promoter element around -30 of transcription start
**TC-rich repeats**	928(+)	ATTTTCTTCA	cis-acting element involved in defense and stress responsiveness
**WUN-motif**	808(-)	AAATTTCCT	wound-responsive element
**Circadian**	618(+)	CAAAGATATC	cis-acting regulatory element involved in circadian control
**CACG**	364(+),427(+), 681(+),1152(+), 1190(+), 1547(+)	CACG	core binding site of NAC factor domain
